# Supplementary material for: The interplay of economic shocks and cultural practices in child marriage: Comparative evidence from India and Zambia during the COVID-19 pandemic
Source: PLoS One. 2026 Jun 3;21(6):e0346851. doi: 10.1371/journal.pone.0346851 (PMC13232802; doi:10.1371/journal.pone.0346851)
Supplement: S1 File — (PDF) [file pone.0346851.s006.pdf]

Inclusivity in global research

PLOS' policy on inclusivity in global research aims to improve transparency in the reporting of research performed outside of researchers' own country or community and ensures that PLOS publications reporting global research adhere to high standards for research ethics and authorship. Authors of relevant research articles may be asked to complete the questionnaire below, which outlines ethical, cultural, and scientific considerations specific to inclusivity in global research. This questionnaire may be requested when researchers have travelled to a different country to conduct research, if research uses samples collected in another country, research with Indigenous populations or their lands, or if research is on cultural artefacts. Researchers travelling to another country solely to use laboratory equipment will not normally be required to complete the questionnaire. However, the questionnaire can be requested at the journal's discretion for any submission – if you have been requested to complete this questionnaire by the PLOS journal you submitted to, please do so.

Please complete the questionnaire below and include this as a Supporting Information file with your manuscript. Note that if your paper is accepted for publication, this checklist will be published with your article in the supporting information files. Please ensure that you reference the checklist in the main body of your manuscript. We suggest adding a subsection 'Inclusivity in global research' to your Methods section and adding the following sentence: "Additional information regarding the ethical, cultural, and scientific considerations specific to inclusivity in global research is included in the Supporting Information (SX Checklist)"

The questions have been designed to be applicable to a wide range of study types, and there are subsections for both human subjects research and non-human subjects research. If any of the questions are not relevant to your research please mark them as "N/A" as appropriate.

Ethical considerations, permits and authorship

This section is applicable to all research types.

Provide details as to who granted permissions and/or consent for the study to take place in the Methods section of your manuscript. This should include the names of **all** ethics boards, governmental organizations, community leaders or other bodies that provided approval for the study. If individuals provided approval refer to these people by their role or title but do not list their name(s).

Reported on page number: 16 (note that we here report page numbers of the marked-up version)<br>"In India, the study was approved by the ethics committee of the Indian Institute of Technology Gandhinagar (IITG) in November 2021. In Zambia, the study was approved by the National Health Research Authority in July 2022. Both study components were additionally approved by the ethics committee of the medical faculty at the Technical University of Munich (TUM) on 29 December 2021."

If there were any deviations from the study protocol after approval was obtained please provide details of these changes in the Methods section of your manuscript.

Reported on page number: NA

Did this study involve local collaborators that are residents of the country where the research was conducted or members of the community studied? If you do not have any authors from said communities, please provide an explanation for this below.

For India, Ms. Shruti Shukla is an Indian national who is pursuing her PhD in Germany at the moment. She is fluent in Marathi and Hindi and has led the enumerator training and data collection in India on the ground. For Zambia, we had tried to mobilise local co-authors but faced considerable challenges as most individuals we approached were too time constrained and had no capacity to contribute to the manuscript in line with the PLOS' criteria for authorship, also due to a lack of incentives for scientific co-authorship from their institutions and employers. However, we have made sure that all involved team members are acknowledged by name in our acknowledgement section: "*For data collected in Zambia, we wish to thank the team from IPSOS, including Oscar Mutinda, Tukiya Mbewe, and Winnie Sambu, as well as our other project partners Alisha Myers, Lawrence Banda, and Ana Garcia Hernandez. We are also deeply grateful to our enumerators: Oness Hinamanjolo, Adinator Mukonka, Sarah Mapiki, Nalucha Sikananu, Mable Mukulumwa, Chilwana Chilenga, Mamire Hamiyanda, Brenda Nkolola, Nachamba Chipompwe, Pumulo Njani, Cynthia Choongo, Cynthia Handili, Namwinga Himonga, Martha Kayawe, Latoya Ntambu, Linety Siagwelele, Annie Masheke, Luyando Himonga, Zithe Mwale, and Monde Masheke.*"

Everyone listed as an author should meet PLOS' criteria for authorship and all individuals who meet these criteria should be included in the author byline, rather than the acknowledgements. For further information please see the journal's Authorship Policy.

Human subjects research (e.g. health research, medical research, cross-cultural psychology)

Did you obtain written informed consent from a representative of the local community or region before the research took place? How did you establish who speaks for the community? Details of written informed consent obtained from study participants should be reported separately in the Methods section of your manuscript.

Yes, see p. 14: "In both countries, we sought written (or finger printed, in the case of limited literacy) informed consent from girls who were 18 years or above. For girls under the age of 18 years (the majority of our sample), we adopted a two-stage consent procedure by first collecting written informed consent from a parent or legal guardian and subsequently collecting verbal assent from each girl."

How did members of the local community provide input on the aims of the research investigation, its methodology, and its anticipated outcome(s)?

We received ethics approval from local IRBs in both Zambia and India, where the consent forms translated to the local languages were carefully reviewed. In addition, consent forms were extensively discussed and edited during our enumerator trainings, making sure that they were easily understandable for possibly low-literacy adolescents in our study settings. Enumerators also received detailed guidance on how to verbally explain consent forms and check for participants' understanding before taking their fingerprints/signatures. This was also practiced through role plays and joint feedback sessions.

When engaging with the local community, how did you ensure that the informed consent documents and other materials could be understood by local stakeholders?

In both settings, enumerators represent the local communities and contributed to the research through in-depth feedback on the questionnaire and the translation and cultural meaning of the individual questions. In addition, in both settings, we presented study findings and interpretations to the enumerator teams and received valuable feedback, which led to additional refinements of our interpretations.

Will the findings of the research be made available in an understandable format to stakeholders in the community where the study was conducted (e.g. via a presentation, summary report, copies of publications, etc.)? Please provide details of how this will be achieved.

Yes. We have written up policy briefs in lay language, which we have shared with our policy partners (SNEH Foundation and ASTITVA in India and World Bicycle Relief in Zambia). Insights from our research have also been shared via social media posts by our partner organisations. In addition, we have organized debriefing sessions with the local study teams and have presented our research findings and asked for additional input from their side.

Non-human subjects research using specimens/ animals collected as part of the study, or those housed in archival collections. Examples include archaeology, paleontology, botany and zoology.

Did the permission you obtained from a local authority to perform the study include an agreement on access to outputs and benefit sharing? This may include procedures to enable fair distribution of the benefits and resources arising from the research performed. Please include any details of Prior Informed Consent and Benefit Sharing Agreements obtained. These may be required by field-specific regulations, for example the Convention on Biological Diversity (CBD) and the associated Nagoya Protocol.

NA

If the material used in your study was imported, please A) provide the year it was imported and B) indicate whether permits were obtained to import/export the materials used, C) provide details of any permits obtained. If this information is not available, please indicate this.

NA

If you used archival specimens, please state how the material used in your study was acquired by the institute it is held in and provide details of any permits obtained for the original excavations/ sample collection. If this information is not available, please indicate this.

NA

How was the potential cultural significance of the materials collected in your study to local communities considered in your research design? Were Indigenous peoples and/or local researchers and institutions involved with archaeological excavations / collection of specimens? If so, please provide a description of their involvement.

NA

If your manuscript includes photographs of human remains please indicate whether authors obtained permission from descendants or affiliated cultural communities to do so.

NA
