# Supplementary material for: The interplay of economic shocks and cultural practices in child marriage: Comparative evidence from India and Zambia during the COVID-19 pandemic
Source: PLoS One. 2026 Jun 3;21(6):e0346851. doi: 10.1371/journal.pone.0346851 (PMC13232802; doi:10.1371/journal.pone.0346851)
Supplement: Attachment — Submitted filename: PONE-D-25-40714_reviewer.pdf [file pone.0346851.s008.pdf]

PLOS ONE

The Interplay of Economic Shocks and Cultural Practices on Child Marriage: Comparative Evidence from India and Zambia during the COVID-19 Pandemic

--Manuscript Draft--

**Manuscript Number:**	PONE-D-25-40714
**Article Type:**	Research Article
**Full Title:**	The Interplay of Economic Shocks and Cultural Practices on Child Marriage: Comparative Evidence from India and Zambia during the COVID-19 Pandemic
**Short Title:**	Child Marriage in India and Zambia: The COVID-19 Pandemic and Cultural Factors
**Corresponding Author:**	Janina I Steinert, DPhil<br>TUM: Technische Universitat Munchen<br>Goettingen, DEUTSCHLAND GERMANY
**Keywords:**	Child Marriage; Cultural Norms; Economic Shock; COVID-19; Zambia; India
**Abstract:**	Child marriage is a critical issue in many low- and middle-income countries (LMICs), further exacerbated by the COVID-19 pandemic due to disruptions in education and economic instability. This study examines the impact of the pandemic on child marriage rates in India and Zambia, focusing specifically on socio-cultural and economic influences. We collected qualitative and quantitative data from adolescent girls aged 13-18 years in India (n=3049) and 15-19 years in Zambia (n=1615) between February and September 2022. Multivariable linear probability regression analyses were applied to assess the pandemic's effect on child marriage and how it was moderated by cultural practices of bride price (Zambia) and dowry (India). Thematic analysis of qualitative data provided further insights into the drivers behind child marriages. While we found no significant increase related to pandemic-induced economic strains in Zambia, we observed a significant increase in child marriage rates related to the pandemic circumstances in India. Qualitative data indicated that reduced dowry demands and lower wedding costs drove families to marry off their daughters during the pandemic. Our findings suggest that economic or health shocks affect child marriage practices differently based on local socio-cultural context and financial practices.
**Order of Authors:**	Pascal Mohamed Mounchid
	Shruti Shukla
	Janina I Steinert, DPhil
**Additional Information:**	
**Question**	**Response**
**Financial Disclosure**<br><br>Enter a financial disclosure statement that describes the sources of funding for the work included in this submission. Review the submission guidelines for detailed requirements. View published research articles from *PLOS ONE* for specific examples.<br><br>This statement is required for submission and **will appear in the published article** if the submission is accepted. Please make sure it is accurate.	Yes

Powered by Editorial Manager® and ProduXion Manager® from Aries Systems Corporation

**Funded studies**<br>Enter a statement with the following details:<br>• Initials of the authors who received each award<br>• Grant numbers awarded to each author<br>• The full name of each funder<br>• URL of each funder website<br>• Did the sponsors or funders play any role in the study design, data collection and analysis, decision to publish, or preparation of the manuscript?<br><br>Did you receive funding for this work?	
Please add funding details.<br>as follow-up to "**Financial Disclosure**<br><br>Enter a financial disclosure statement that describes the sources of funding for the work included in this submission. Review the submission guidelines for detailed requirements. View published research articles from *PLOS ONE* for specific examples.<br><br>This statement is required for submission and **will appear in the published article** if the submission is accepted. Please make sure it is accurate.<br><br>**Funded studies**<br>Enter a statement with the following details:<br>• Initials of the authors who received each award<br>• Grant numbers awarded to each author<br>• The full name of each funder<br>• URL of each funder website<br>• Did the sponsors or funders play any role in the study design, data collection and analysis, decision to publish, or preparation of the manuscript?<br><br>Did you receive funding for this work?"	German Research Foundation
Please select the country of your main research funder (please select carefully as in some cases this is used in fee	GERMANY - DE

calculation).<br>as follow-up to "**Financial Disclosure**<br><br>Enter a financial disclosure statement that describes the sources of funding for the work included in this submission. Review the submission guidelines for detailed requirements. View published research articles from *PLOS ONE* for specific examples.<br><br>This statement is required for submission and **will appear in the published article** if the submission is accepted. Please make sure it is accurate.<br><br>**Funded studies**<br>Enter a statement with the following details:<br>• Initials of the authors who received each award<br>• Grant numbers awarded to each author<br>• The full name of each funder<br>• URL of each funder website<br>• Did the sponsors or funders play any role in the study design, data collection and analysis, decision to publish, or preparation of the manuscript?<br><br>Did you receive funding for this work?"	
**Competing Interests**<br><br>Use the instructions below to enter a competing interest statement for this submission. On behalf of all authors, disclose any competing interests that could be perceived to bias this work—acknowledging all financial support and any other relevant financial or non-financial competing interests.<br><br>This statement is required for submission and **will appear in the published article** if the submission is accepted. Please make sure it is accurate and that any funding sources listed in your Funding Information later in the submission form are also declared in your Financial Disclosure	The authors have declared that no competing interests exist.

Powered by Editorial Manager® and ProduXion Manager® from Aries Systems Corporation

statement.<br><br>View published research articles from *PLOS ONE* for specific examples.<br><br>**NO authors have competing interests**<br><br>Enter: *The authors have declared that no competing interests exist.*<br><br>**Authors with competing interests**<br><br>Enter competing interest details beginning with this statement:<br><br>*I have read the journal's policy and the authors of this manuscript have the following competing interests: [insert competing interests here]*<br><br>* typeset	
**Ethics Statement**<br><br>Enter an ethics statement for this submission. This statement is required if the study involved:<br><br>• Human participants<br>• Human specimens or tissue<br>• Vertebrate animals or cephalopods<br>• Vertebrate embryos or tissues<br>• Field research<br><br>Write "N/A" if the submission does not require an ethics statement.<br><br>General guidance is provided below. Consult the submission guidelines for detailed instructions. **Make sure that all information entered here is included in the Methods section of the manuscript.**	In India, the study was approved by the ethics committee of the Indian Institute of Technology Gandhinagar (IITG) in November 2021. In Zambia, the study was approved by the National Health Research Authority in July 2022. Both study components were additionally approved by the ethics committee of the medical faculty at the Technical University of Munich (TUM) on 29 December 2021

**Format for specific study types**<br><br>**Human Subject Research (involving human participants and/or tissue)**<br>• Give the name of the institutional review board or ethics committee that approved the study<br>• Include the approval number and/or a statement indicating approval of this research<br>• Indicate the form of consent obtained (written/oral) or the reason that consent was not obtained (e.g. the data were analyzed anonymously)<br><br>**Animal Research (involving vertebrate animals, embryos or tissues)**<br>• Provide the name of the Institutional Animal Care and Use Committee (IACUC) or other relevant ethics board that reviewed the study protocol, and indicate whether they approved this research or granted a formal waiver of ethical approval<br>• Include an approval number if one was obtained<br>• If the study involved *non-human primates*, add *additional details* about animal welfare and steps taken to ameliorate suffering<br>• If anesthesia, euthanasia, or any kind of animal sacrifice is part of the study, include briefly which substances and/or methods were applied<br><br>**Field Research**<br><br>Include the following details if this study involves the collection of plant, animal, or other materials from a natural setting:<br>• Field permit number<br>• Name of the institution or relevant body that granted permission	
**Data Availability**<br><br>Authors are required to make all data underlying the findings described fully available, without restriction, and from the time of publication. PLOS allows rare exceptions to address legal and ethical concerns. See the PLOS Data Policy and FAQ for detailed information.	Yes - all data are fully available without restriction

A Data Availability Statement describing where the data can be found is required at submission. Your answers to this question constitute the Data Availability Statement and **will be published in the article**, if accepted.<br><br>**Important:** Stating 'data available on request from the author' is not sufficient. If your data are only available upon request, select 'No' for the first question and explain your exceptional situation in the text box.<br><br>Do the authors confirm that all data underlying the findings described in their manuscript are fully available without restriction?	
**Describe where the data may be found in full sentences. If you are copying our sample text, replace any instances of XXX with the appropriate details.**<br><br>• If the data are **held or will be held in a public repository**, include URLs, accession numbers or DOIs. If this information will only be available after acceptance, indicate this by ticking the box below. For example: *All XXX files are available from the XXX database (accession number(s) XXX, XXX.)*.<br>• If the data are all contained **within the manuscript and/or Supporting Information files**, enter the following: *All relevant data are within the manuscript and its Supporting Information files.*<br>• If neither of these applies but you are able to provide **details of access elsewhere**, with or without limitations, please do so. For example:<br><br>*Data cannot be shared publicly because of [XXX]. Data are available from the XXX Institutional Data Access / Ethics Committee (contact via XXX) for researchers who meet the criteria for access to confidential data.*<br><br>*The data underlying the results presented in the study are available from (include the name of the third party*	All data and code is available via https://osf.io/2xgpb/

Powered by Editorial Manager® and ProduXion Manager® from Aries Systems Corporation

and contact information or URL).<br>• This text is appropriate if the data are owned by a third party and authors do not have permission to share the data.<br><br>* typeset	
Additional data availability information:	Tick here if the URLs/accession numbers/DOIs will be available only after acceptance of the manuscript for publication so that we can ensure their inclusion before publication.

Powered by Editorial Manager® and ProduXion Manager® from Aries Systems Corporation

Manuscript

Click here to access/download;Manuscript;Child_Marriage_and_COVID_Manu

The Interplay of Economic Shocks and Cultural Practices on Child Marriage: Comparative Evidence from India and Zambia during the COVID-19 Pandemic *

Pascal Mohamed Mounchid† Shruti Shukla‡ Janina Isabel Steinert§

July 30, 2025

Abstract

Child marriage is a critical issue in many low- and middle-income countries (LMICs), further exacerbated by the COVID-19 pandemic due to disruptions in education and economic instability. This study examines the impact of the pandemic on child marriage rates in India and Zambia, focusing specifically on socio-cultural and economic influences. We collected qualitative and quantitative data from adolescent girls aged 13-18 years in India (n=3049) and 15-19 years in Zambia (n=1615) between February and September 2022. Multivariable linear probability regression analyses were applied to assess the pandemic's effect on child marriage and how it was moderated by cultural practices of bride price (Zambia) and dowry (India). Thematic analysis of qualitative data provided further insights into the drivers behind child marriages. While we found no significant increase related to pandemic-induced economic strains in Zambia, we observed a significant increase in child marriage rates related to the pandemic circumstances in India. Qualitative data indicated that reduced dowry demands and lower wedding costs drove families to marry off their daughters during the pandemic. Our findings suggest that economic or health shocks affect child marriage practices differently based on local socio-cultural context and financial practices.

Keywords: Child Marriage, Cultural Norms, Economic Shock, COVID-19, Zambia, India

*We are grateful for funding received from the German Research Foundation, and World Bicycle Relief. For data collected in India, we wish to thank Rucha Vasumati Satish, the SNEH Foundation and ASTITVA Foundation as well as our dedicated team of enumerators: Vandana Murlidhar Kamble, Jayshree Kamble, Fairaza Mulla, Manali Amol Vhankade, Madhuri Malhari Mandale, Kajal Vitthal Garale, Shivani Aapte, Rajashree Nitin Vhankade, Deepa Ramhari Bhusanar, Sunita Gadhire, Sonal Umesh Bhoyar, Priyanka Raju Chandane, Nafisabanu Farid Shaikh, Rashmi Adinath Khandagale, Vimal Hemant Pise, Savita Ramdas Durge, Sunita Salunke, Dorthi Robin Joseph, Aruna Hitendra Kamble, Pallavi Abasaheb Sangale, Jyoti Lahurao Surywanshi, Shubhangi Sachin Salve, Bhagyashree Patole, Shraddha Bhargande, Samina Shaikh, and Shilpa Samant.For data collected in Zambia, we wish to thank Alisha Myers, Lawrence Banda, Oscar Mutinda, Tukiya Mbewe, Winnie Sambu, and Ana Garcia Hernandez. We also wish to think all girls who volunteered their time to participate in this research study and shared their stories with us.

†University of Konstanz. pascal.mounchid@uni-konstanz.de.

‡Technical University of Munich. shruti.shukla@tum.de.

§Technical University of Munich. janina.steinert@tum.de. *Corresponding Author*, Richard Wagner Str. 1, 80333 Munich

1 Introduction

Child marriage is a global problem rooted in gender inequalities and the belief that the social status of girls and women is inferior to their male counterparts. It is defined as "any formal marriage or informal union between a child under 18 and an adult or another child" (UNICEF, 2024). [1] Given the detrimental impacts of child marriage on the health and wellbeing of affected children, the United Nations Sustainable Development Goal 5.3 aims to eradicate child marriage by 2030.

Girls are six times more likely than boys to be married before the age of 18 (Hassfurter, 2021). UNICEF estimates that approximately one in five girls worldwide are married during childhood (UNICEF, 2024). The highest rates of child marriage are found in Sub-Saharan Africa, with 34 per cent, and in South Asia, with 28 per cent of young women married in childhood (United Nations Children´s Fund, 2021). For these girls, marriage often marks the abrupt discontinuation of their education, long-time economic dependence on their spouses, an increased risk of facing partner abuse, and a higher likelihood of adolescent pregnancy, despite the associated health risks for both the mother and child (Carrico et al., 2020; Corno et al., 2020; Field and Ambrus, 2008; Godha et al., 2013; Raj et al., 2009). While the overall trend of child marriages is declining, scholars have raised concerns about a possible surge in child marriages due to the COVID-19 pandemic (Hossain et al., 2021; Musa et al., 2021; Rahiem, 2021). Specifically, a Lancet publication has warned that the COVID-19 pandemic may put an additional 2.5 million girls around the world at risk of child marriage within the next five years (Cousins, 2020).

The circumstances leading to child marriages are complex, involving a blend of more static factors, such as a family's socio-demographic background and their social and patriarchal norms, and dynamic factors, such as economic shocks and unforeseen emergencies, such as a pandemic. For example, the bride's family might aim to marry off their daughter as soon as she reaches puberty, both for moral reasons and to protect her from potential sexual assault and harassment by other men, which could significantly reduce her marriage prospects and harm the family's honour or social status in the long term (Alston et al., 2014; Caldwell et al., 1998). Further, marriage decisions can be driven by economic considerations, particularly when families face financial shortages and economic pressures. In patrilocal contexts, where it is customary for the bride to move in with her family-in-law after marriage, a daughter's marriage can serve as a coping mechanism, as scarce financial resources

[1] In a few studies, the term "early marriage" has also been used, with the marriage age of women being defined as under 18 and that of men as under 21 (Alston et al., 2014). However, in the current paper, we will use the term "child marriage" throughout.

and food in her natal family would then need to be shared with one household member less (Ahmed et al., 2019; Dietrich et al., 2022). Another economic motivation for marrying off a daughter may be the receipt of a bride price - a transfer from the groom's family to the bride's family, a common tradition in many Sub-Saharan African countries. This can become a consumption smoothing strategy for families under immense economic pressure to balance their current spending and savings for the future. For example, a qualitative study on child marriage in Zambia highlights that many parents and guardians use child marriage as an opportunity to benefit financially from the bride price to ease the strain of household resources and to free up resources to support younger children (Population Council, UNFPA, 2024).

While previous studies have extensively analyzed how external economic shocks, particularly those from natural disasters, affect child marriage rates (Corno et al., 2020; Dietrich et al., 2022; Trinh and Zhang, 2021), our understanding remains limited regarding how variations in marriage customs—such as bride price versus dowry systems—and differing gender norms across countries might moderate these effects. Research has yet to thoroughly investigate how these cultural and economic factors interact to influence child marriage risk during the COVID-19 pandemic. Recent pandemic-related studies have begun illuminating these connections, so Hofisi and Hungwe (2025) examined the Zimbabwe's context and show a string decrease in women´s ability to get education while because of own pregnancy. Further, Chort et al. (2025) documented increased marriage trends in Turkey, building on work by Jibon et al. (2025) and Julianto et al. (2025). However, as Haq et al. (2024)[p.9] argue, "mixed studies combining qualitative and quantitative methods are needed to examine the effects of the pandemic on marriage." This paper addresses these research gaps by examining: (i) whether the economic burden imposed by COVID-19 increased child marriage risk, and (ii) whether this effect differs between bride price contexts, where resources transfer from groom's to bride's family, and dowry contexts, where transfers flow in reverse.

Our study provides the first comparative analysis between India and Zambia, combining quantitative regression techniques with interviews of young women to capture the complete picture of pandemic impacts on child marriage practices. We used this two countries, because they do not only to distinct marriage customs, —India being a dowry-based society and Zambia practicing bride prices—but also belong to regions with some of the highest rates of child marriage globally. Although the data presented in the following tables may suggest a gradual decline in child marriage rates, the absolute numbers remain alarmingly high (Child Marriage Data Portal, 2025). In India, the situation is partic-

ularly concerning: an estimated 4,442 girls are married every day—equivalent to approximately three girls every minute—resulting in over 1.6 million child brides annually (India Child Protection, 2025).

Table 1: Child Marriage Rates in India by Year

1996	2001	2006	2011	2016	2021
53.2%	48.8%	45.7%	38.0%	29.5%	23.3%

For Zambia, the currently available data indicate a downward trend in child marriage rates over the past decades. As shown in Table 2, the rate has decreased from 46.9% in 1993 to 29% in 2018.

Table 2: Child Marriage Rates in Zambia by Year

1993	1998	2003	2008	2013	2018
46.9%	42.7%	42.8%	38.2%	32.4%	29.0%

While this long-term progress in both countries is encouraging, there is currently a lack of comprehensive nationally representative data that would allow for a fine-grained assessment of the impact of the COVID-19 pandemic on child marriage rates, which may have significantly disrupted this positive development. According to Equality Now (2020), the pandemic has exacerbated many of the underlying drivers of child marriage across Africa—such as poverty, school closures, and reduced access to protection services, which could drive surges in underage marriages.

This paper contributes to the existing literature by exploring the nuanced effects of the COVID-19 pandemic on child marriage rates, considering the economic burdens imposed by the pandemic and how local marriage customs may mediate these. The findings will offer insights into how different cultural and economic contexts influence the risk of child marriage, informing policymakers and stakeholders about specific vulnerabilities and mechanisms at play.

The paper proceeds as follows. The next section introduces the literature on the factors influencing child marriage and presents the theoretical framework on how the COVID-19 pandemic and cultural factors may have affected child marriage rates in two selected low- and middle-income countries: India and Zambia. In the subsequent section, we describe the study sample and data collection and outline the analysis strategy. The following section presents the descriptive and analytical findings and the paper concludes with a discussion of wider policy and research implications.

2 Theoretical Background and Related Literature

To guide our analysis, we create a conceptual framework that draws upon the model proposed by Kok et al. (2023), which identifies four essential levels that contribute to the occurrence of child marriages, namely the (i) individual, (ii) social, (iii) material, and (iv) institutional level (Kok et al., 2023, p. 8). The *individual level* comprises personal characteristics like age, educational attainment, employment and marriage knowledge of girls and young women. The parents' educational background, household size and the family's status in the community are attributed to the *social level*. The *material level* pertains to the family's economic status, whereas the *institutional level* encapsulates the significance of the legal system, societal norms and cultural values and beliefs on gender roles. In this paper, the social and the material level are combined into one *socio-economic level* to streamline the complexity of dimensions while ensuring the inclusion of pertinent micro-mechanisms. Additionally, we extend the framework by Kok et al. (2023) with one additional factor: *external shocks*, which will be introduced to assess the impact of the COVID-19 pandemic on the likelihood of child marriage to occur.

Correlates of child marriage at the individual and socio-economic level

Concerning the socio-economic conditions that increase the likelihood of child marriage, several studies have identified the parents' and the girls' level of education as strong predictors (Alem et al., 2020; Ali et al., 2014; Al-Ridhwany and Al-Jawadi, 2014; Aychiluhm et al., 2021). For example, Ali et al. (2014) show in their analysis of survey data from Sudan that women who have only acquired primary education are significantly more likely to be married under 18 than those with a higher level of education. It is also known that child marriages particularly often affect illiterate girls (Dharan and Sah, 2014). Apart from this, Envuladu et al. (2016) emphasise that a low level of education among parents increases the likelihood of their daughter being married underage. At the same time, the authors point out that the economic status of the family is equally important as they observe higher marriage rates among girls whose fathers were farmers, thus relying on lower and more seasonal incomes (Envuladu et al., 2016, p.125). Recent evidence from Bangladesh shows that financial incentives can significantly reduce child marriage, whereas empowerment programs alone may be insufficient. The findings suggest that early marriage may serve as a strategic signal in the marriage market, especially where traditional norms dominate. In such contexts, altering economic incentives appears more effective than attempting to shift gender norms directly (Buchmann et al., 2023). In addition to Akter et al. (2021) and Hoogeveen et al. (2011), the study by Kok et al. (2023) points to the exceptionally high density of child marriages in poorer regions. Based on the potent effect of poverty, which can

occur particularly strongly in rural areas (Envuladu et al., 2016), Al-Ridhwany and Al-Jawadi (2014) confirm an urban-rural divide in child marriage rates in their study from Northern Iraq. Corroborating this finding with data from Ethiopia, Aychiluhm et al. (2021) show that child marriages take place particularly in rural and impoverished areas of the country. Another study from Ethiopia also explores the links between household wealth and child marriages, revealing that particularly assets owned by a mother are associated with lower rate of girls' marriage rates, whereas paternal assets do not seem to be a key factor (Muchomba, 2021).

Another significant social factor contributing to the prevalence of child marriage rates is parental authority. Envuladu et al. (2016) describe that parents or guardians may coerce their children into early marriage, potentially motivated by a desire to alleviate the financial responsibilities associated with raising their offspring and instead shift them onto another family. Additionally, Aychiluhm et al. (2021) argues that child marriages are frequently arranged in contexts of limited autonomy of the prospective bride and substantial pressure exerted by her parents.

Correlates of child marriage on the institutional level

The law around child marriage differs from country to country but often targets the minimum age of marriage and informed consent. To protect girls and boys under the age of 18, countries may adopt various strategies like criminalization of child marriages, banning marriage below the legally prescribed minimum age, or prescribing a minimum age of marriage but not criminalizing or banning it (United Nations Population Fund, 2020). For example, under the Indian Prohibition of Child Marriage Act of 2006, where the minimum age of marriage for girls is 18 and boys is 21, people involved in performing, conducting, or directing a child marriage can be imprisoned. Further, child marriages can be nullified within two years of the minor reaching adulthood (Government India, 2006). In Zambia, while the minimum age for marriage is 18 years under the Prohibition of Child Marriage Act, individuals between 16 and 21 could get married with parental consent until 2023. In addition, customary laws often took precedence over statutory law, making enforcement of the minimum age difficult (Population Council, UNFPA, 2024; Equality now, 2024). However, previous studies have shown that laws have a limited impact on child marriage prevalence, and they need to be integrated into broader gender transformative approaches that tackle underlying causes like gender norms, low status of women and girls and cultural values and beliefs (Collin and Talbot, 2017; Girls not brides, 2024; Wilson, 2022).

Fattah and Camellia (2022) conducted interviews with young girls in Bangladesh, revealing their profound awareness of the adverse ramifications of early marriage. Despite this, parental approval of

child marriage persists, purportedly, because married girls are seen as imposing a lower social stigma on the family's status in the event of sexual assault or harassment. The belief that the unmarried status of young girls tarnishes the family's reputation following an attack is evident across various regions, including North African countries (Kohno et al., 2020), Ethiopia, Ghana (Ahonsi et al., 2019; Psaki et al., 2021) and India (Corno et al., 2020). Such entrenched value systems appear to be a crucial barrier to the success (or effectiveness) of interventions that seek to enhance the self-determination of young girls (Pourtaheri et al., 2023). Traditional gender roles thus predetermine the trajectories of girls' lives, with relationships grounded in partnership occasionally being achievable solely through marriage. Furthermore, the societal expectation for women in these contexts is to assume roles as "passive, abnegated wives and mothers" (Pacheco-Montoya et al., 2022, p.26). The prospect of abstaining from child marriage appears scarcely desirable, as besides the pressure exerted by the social milieu surrounding young girls (Abdurahman et al., 2023), the entire family would face societal sanctions and ostracism (Steinhaus et al., 2019; Tewahido et al., 2022). Moreover, religious convictions may also impede the expansion of freedoms for young girls. For instance, (Ahonsi et al., 2019, p.2) underscore that major religious traditions in Ghana encourage early marriage. However, it is essential to note that a universal impact of religious tenets cannot be assumed as two studies by Cislaghi et al. (2019, 2020) from Cameroon elucidate that certain traditions permit marriage only upon reaching the age of majority, thus fulfilling the obligation of marriage.

The influence of external shocks on child marriage

A body of literature has examined how external shocks, such as those introduced by unforeseen extreme weather events or natural disasters, may affect girls' vulnerability to underage marriage. For example, Ahmed (2018) conducted a study investigating the repercussions of the 2010 flood disaster in Pakistan, finding that there was a significant decrease in child marriage rates contrary to the authors' initial expectations. The authors contended that the calamity likely engendered a state of emergency that compelled individuals from forming partnerships as a means of emotional coping [2]. However, this observation's generalisability was challenged by a subsequent study by Ahmed et al. (2019), which investigated the impact of a flood disaster in Bangladesh. The researchers highlighted the high poverty rates prevalent in the affected regions and demonstrated an increase in child marriages following the disaster. Drawing on focus group interviews, they argued that parents of married daughters deemed child marriage necessary due to the family's economic constraints stemming from the disaster. Marriage was perceived as a means to transfer the financial responsibility for the daughter to the groom's

[2] Moreover, Ahmed (2018) underscored the possible adherence to legal regulations regarding the minimum age of marriage, notably emphasising the Child Marriage Restraint Act of 1929, which unequivocally prohibits child marriages.

family, thereby alleviating the financial burden the bride's family faced. Other analyses based on samples from India corroborate this observation (Asadullah et al., 2021; Khanna and Kochhar, 2021). Blanco (2023) assesses how exposure to different earthquakes within Indonesian provinces between 1994 and 2014 affected girls' chances of being married under age. She reveals a 44% increase in the annual hazard of girls marrying before the age of 18. In line with previous explanations, the author finds that the daughter's marriage may function as a financial coping strategy through the receipt of a marriage payment. Yet, she also reveals evidence for an additional mechanism, whereby the destruction of school buildings caused a drop in school attendance, consequently increasing marriage rates. Significant increases in child marriage rates following different natural disasters are further shown in a study by Das and Dasgupta (2022), who analysed the societal consequences of the 2001 Gujarat earthquake in India, as well as in studies by Tsaneva (2020) and Carrico et al. (2020) who assess the impact of heat waves in South Asia. Carrico et al. (2020) highlight that increasing child marriage rates can also persist over time, as "girls and women between the ages of 11 and 23 were at an increased risk of marrying in the year of, or after [heat waves]in Bangladesh" (Carrico et al., 2020, p.13).

Another type of external shock could have been introduced by the COVID-19 pandemic and the linked economic repercussions caused by lockdown measures and considerable employment losses. The particular burden placed on developing countries by the pandemic became apparent early on. In particular, the ability to cushion the economic consequences of the necessary lockdown measures was barely possible in these countries. Before the pandemic began, there were warnings that poverty rates would rise in the short and medium term, as previous work could no longer be carried out due to the pandemic measures Bottan et al. (2020). South Asia, where household income was earned through labour migration, was particularly hard hit by travel and movement restrictions (Buheji et al., 2020). Household income was also severely reduced in African countries due to the pandemic, and poverty rates rose significantly in a short period (Bundervoet et al., 2022; Diop and Asongu, 2023; Miguel and Mobarak, 2022). Empirical evidence on how the COVID-19 pandemic has affected child marriage rates is still scarce or is methodological thin. One study by Melnikas et al. (2023) draws on data from repeated cross-sectional surveys that were conducted as part of the "More Than Brides Alliance" impact evaluation in 609 villages across four Indian states. Observing child marriage rates over the course of five years, the authors are able to show declines in marriage rates between their baseline and midline measurement, which occurred prior to the COVID-19 pandemic, but then found that this trend stagnated in the course of the pandemic and that child marriage rates increased slightly between midline and endline measurement. Conversely, Batheja et al. (2022) leverage data from the Indian National

Family Health Survey conducted between 2019-2021 (NFHS-5) and reveal a significant increase in the age at marriage when comparing women who got married after the onset of the pandemic to those married before. The authors suggest that the observed decline in child marriage rates may be driven by a negative income shock, which made it impossible for the bride's parents to afford the wedding and dowry payment for their daughter. The dowry is a customary transfer from the bride's family to the groom's family and constitutes a significant economic transaction that operates in the opposite direction of a bride price.

2.1 Research gap

Understanding the full impact of the COVID-19 pandemic and external or economic shocks more broadly, requires attention to cultural practices, such as marriage-related transfers, which shape households' strategies for coping with financial strain. Yet, existing research rarely economic stress with the respective local culture context. For instance, Jibon et al. (2025) document pandemic-related economic stress and rising child marriage rates, but without accounting in detail for customs like dowry or bride price. Similarly, studies such as Julianto et al. (2025) and the review by Haq et al. (2024) highlight economic determinants while neglecting cultural mechanisms. Brandl and Colleran (2024) provide a theoretical link between marriage payments and marriage patterns, but their analysis remains largely conceptual. Our paper uses comparative evidence from India and Zambia, in contexts with contrasting norms (dowry versus bride price) to encode how these mechanism interact with each other. Combining quantitative analyses of our surveys with qualitative interviews, we provide both statistical estimates and in-depth contextual understanding. This mixed-methods approach enables us to uncover the micro-mechanisms that drive child marriage under pandemic-related stress, offering a more comprehensive perspective than previous research. The following section introduces our theoretical framework and outlines the hypotheses guiding our analysis.

COVID-19, marriage customs, and child marriage: Theoretical framework and predictions

To examine how income changes such as those caused by natural disasters or external shocks affect marriage prospects, Corno et al. (2020) introduce an analytical model, that will guide the hypotheses we test in this paper. In their model, the researchers make three fundamental assumptions about child marriage. First, it is assumed that girls contribute less to the household welfare than they consume, whereas this effect is not prevalent in boys, who are considered more able to work. Second, it is as-

sumed that, apart from a financial transaction between the groom's and bride's family that is arranged in the context of the marriage, the bride must move-in with the groom's family to shift the financial situation of both households. Third, the model assumes that there is gender balance in the community, leading to a balance in the marriage market. Based on these three assumptions, the micro-mechanisms of the two effect directions are described below, and hypotheses are formulated. To test the predictions of their model, the authors combine weather data with data from the Demographic and Health Survey (DHS) from Sub-Saharan Africa and India. Their analyses reveal that while periods of drought in African countries significantly increased the probability of child marriages by up to three percentage points on average, a similarly significant but *opposite* effect was found in the sample from India, that is, a decrease in the probability of child marriages. Corno et al. (2020) argued that the heterogeneous findings that they observe between the two regional contexts are driven by a third factor: customary marriage transfers.

Based on these findings, our first hypothesis is focused on the customary marriage transfer in Zambia: the "bride price". In this form of marriage transfer, it is assumed that the bride's family must receive financial compensation as the relocation of their daughter to the household of the groom's family implies that she can produce economic value for her family-in-law in terms of household or paid labor (Corno et al., 2020). Thus, a payment in the form of a bride price is made by the groom's to the bride's family. This payment can be particularly high if the bride is considered young and sexually inexperienced at the time of marriage (Corno and Voena, 2023). If a household's income falls sharply and unexpectedly due to an external shock like a natural disaster, families may have to resort to alternative income generation opportunities, particularly in contexts where poverty rates and economic pressures are already high (Makino et al., 2023). Asadullah et al. (2021, p.964) argue that receiving a bride price can serve as a "consumption-smoothing strategy" for families under economic pressure (Ahmed et al., 2019; Blanco, 2023; Carrico et al., 2020). Thus, in societies where the practice of bride price is common, child marriage rates can be expected to increase in times of economic shocks. We believe that the same mechanism likely holds true in the context of the COVID-19 pandemic and its linked economic pressures and will therefore test the following hypothesis:

- **Hypothesis 1: In the context of Zambia where the "bride price" custom is prevalent and where families are economically burdened by the COVID-19 pandemic families, an increase in child marriages can be expected.**

Our second hypothesis shifts focus to the customary marriage transfer prevalent in India: the

"dowry". A dowry denotes a transfer from the bride's family to the groom's family. Dowry payments are often customary in contexts where female unemployment rates are high and where the daughter-in-law is perceived as placing more of an economic burden on the household she lives in (Aychiluhm et al., 2021). In India, dowry payments may even amount up to several times the annual income of the bride's family (Bloch and Rao, 2002; Menon, 2020). If a household is put under severe economic strain due to an external shock, parents of potential brides are incentivized to suspend or delay marriage arrangements to avoid further financial strain. This prediction is supported by empirical evidence from a panel study conducted in Pakistan. The authors show that in villages where marriage costs are high, early marriage was delayed after households had experienced financial shocks and were therefore more credit constrained (Khan, 2024). Applying this to the context of the COVID-19 pandemic, we therefore test the following hypothesis:

- **Hypothesis 2: In the context of India where the "dowry" custom is prevalent and where families are economically burdened by the COVID-19 pandemic families, a decrease in child marriages can be expected.**

The predictions our paper seeks to test based on the model by Corno et al. (2020) are summarised in Figure 1.

Figure 1: Conceptual framework of child marriage

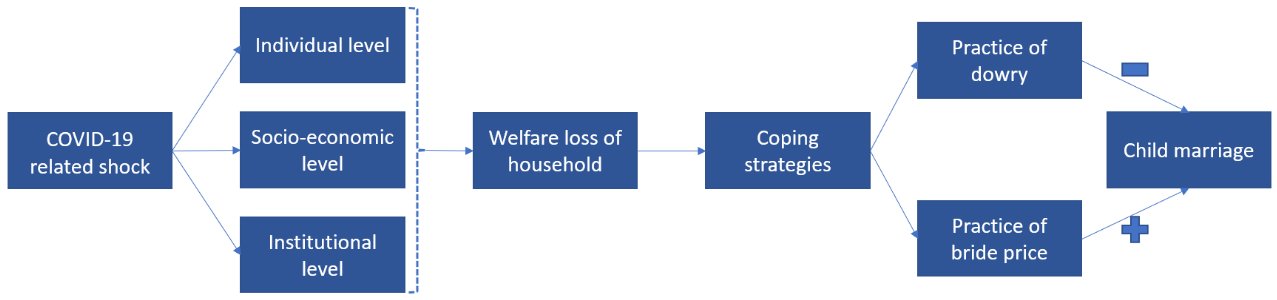

3 Methodology

3.1 Study Setting

The study focuses on two countries: India and Zambia. UNICEF (2024) estimates that 1.5 million girls in India are married before reaching adulthood, and almost one in three girls are married before their 18th birthday in Zambia. Thus, the two countries are adequate settings for examining child marriage rates. More importantly, the selection of these two countries also allows us to investigate the potential impact of differences in marriage customs, considering that the practice of bride price is prevalent in Zambia, whereas Indian society largely relies on the dowry system.

In India, data were collected in the second most populous state of the country, Maharashtra. Specifically, we targeted peri-urban and urban settlements around the city of Pune as well as rural villages around the city of Sangli. In Maharashtra, approximately 25% of the population lives in poverty, with higher rates in rural areas (NITI Aayog, 2021; World Bank, 2018). In Zambia, we collected data in three districts of Zambia's Southern Province: Kalomo, Mazabuka, and Monze. This province is characterized by low population density, many families living in rural settlements, and a high poverty rate, with 51% of households having lived in extreme poverty in 2022 (Population Council, UNFPA, 2024).

3.2 Sampling Strategy

India

In India, we recruited adolescent girls aged 13-18 years. We started with mapping schools and surrounding communities in both study locations, namely Pune and Sangli. School-attending girls were recruited directly within schools where headmasters and class teachers provided a list of all girls aged 13-18 years. Additionally, to recruit girls who had already dropped out of school, the local team conducted door-to-door visits in the surrounding communities to identify girls within the eligible age range who were no longer attending school. This recruitment process was further facilitated by local community leaders and Anganwadi workers using a random walk procedure. Our final sample in India consisted of 3049 girls, of which 1552 had been recruited from urban and peri-urban settlements in Pune and 1497 from rural villages in Sangli.

Zambia

In Zambia, we recruited adolescent girls aged between 15 and 19 years. To identify eligible girls, we

approached 100 schools across the three districts Kalomo, Mazabuka, and Monze to collect information on the surrounding communities from which students typically come to that school. We then randomly selected four communities surrounding each school from this list, aiming to recruit both girls who were still enrolled in school as well as girls who had dropped out of school. Teams of enumerators were sent to these communities to recruit five girls per community, aiming for a final sample size of 20 girls per school cluster. Similarly to our recruitment approach used in India, participants were recruited based on a random walk procedure. Enumerators started from a central location in the community, such as a church or community hall, spanned a pencil, and followed its direction, visiting every third household. Households were screened for eligibility based on whether they had a girl in the household aged between 15 and 19 years. If a household did not meet the eligibility criteria, the enumerators were prompted to continue to the next household according to the random walk rule until the target of five girls per community was achieved. Our final sample consisted of 1615 girls who were recruited within 92 school clusters.[3]

3.3 Data Collection

We collected quantitative data via standardized questionnaires administered on tablets with the assistance of female enumerators. Enumerators in India were recruited via two partner NGOs[4] and were fluent in Marathi and Hindi. Data collection was conducted from 1-30 March 2022. In Zambia, enumerators were recruited by the survey firm IPSOS and were fluent in English and Tonga. Data collection took place between 9 May and 9 December 2022. In both countries, enumerators were extensively trained in interview techniques for collecting sensitive data and research ethics, including informed consent procedures, confidentiality, and referral of at-risk participants. Each interview lasted between 60 and 90 minutes and was conducted at participants' homes, on school grounds after lessons (for girls still attending school) or in a convenient community location (such as a community hall), ensuring that confidentiality could be guaranteed in the setting. Similar to previous studies conducted with adolescents in low-literacy populations (Steinert et al., 2023), the surveys were designed as audio- and computer-assisted self-interviews (ACASI) with the intention of maximising confidentiality as well as reducing possible social desirability bias and under-reporting. In both countries, we sought written (or fingerprinted, in the case of limited literacy) informed consent from girls who were 18

[3]While we had initially planned to cover 100 school clusters, data collection took longer and was more costly than expected since participants' houses were quite spread out, thus requiring long travel distances from one community to the next. Our data collection was terminated prior to reaching the original target sample size of 2000 due to budgetary constraints.

[4]We collaborated with the SNEH Foundation in Pune and the Astitva Foundation in Sangli.

years or above. For girls under the age of 18 years (the majority of our sample), we adopted a two-stage consent procedure by first collecting written informed consent from a parent or legal guardian and subsequently collecting verbal assent from each girl.

In addition to quantitative data, we collected qualitative data. In India, we collected extensive qualitative data from a total of eight focus group discussions (FGDs) and nine individual in-depth interviews. The focus group discussions were carried out prior to the quantitative data collection with groups of mothers (2), fathers (2), adolescent girls (2), and social workers (2). In-depth interviews were conducted after completion of the quantitative survey, and participants were sampled purposely based on having disclosed any of the following adverse outcomes: (i) having experienced family or intimate partner violence in the previous year, (ii) having been married underage, and (iii) reporting a current or past pregnancy. Interview guides were aimed at eliciting the harmful impacts of the COVID-19 pandemic and at discussing their underlying causes. In Zambia, due to budget constraints, we could only conduct a total of three FGDs. These were carried out prior to the quantitative data collection with one group of mothers and two groups of adolescent girls. Similar to the FGDs in India, discussion guides were focused on the harmful impacts of the COVID-19 pandemic. All discussions and interviews were conducted in the respective local language (i.e., Marathi, Hindi or Tonga) and recorded verbatim and then transcribed and translated into English.

3.4 Measures

Dependent Variable

The dependent variable of interest is the girls' marital status, which was dummy-coded as 1 if the girl reported being currently married, divorced or widowed and if the wedding ceremony had occurred before the girl had reached her 18th birthday.[5]

Explanatory Variables

We included explanatory variables reflecting the different levels introduced by Kok et al.'s 2023. theoretical framework outlined above. On the individual level, we included (i) girls' age and (ii) caste (for the Indian sample only).[6] On the socio-economic level, we included the (i) education status of both

[5]The survey asked girls to indicate the date of their wedding ceremony, which was then cross-checked against their age information to ensure that only marriages that had occurred before their 18th birthday were included.

[6]We decided not to include girls' educational status due to endogeneity concerns, given that school dropout could likely be a consequence of child marriage rather than a predictor (see, for example: (Delprato et al., 2017)) and that our cross-sectional data did not allow us to differentiate between cause and consequence.

the mother and father, defined as a binary variable that was coded as 1 if the mother/father reported having completed secondary school; and (ii) households' wealth status, which was an additive index capturing the amount of assets owned by a household as well as achieved living standard indicators such as electricity, flush toilet, and drinking water in the house (see Table A1 for a list of individual indicators of included aggregate measures). On the institutional level, we sought to measure gender norms by including an additive index that measured girls' approval of violence against women, whereby a higher index score denoted a higher acceptance of violence. Specifically, respondents had to state whether they agreed or disagreed with seven different statements asking about scenarios in which it would be justifiable for a husband to beat his wife, for example, if the wife *"goes out without telling her husband"* or *"hides money from her husband"*. Lastly, we included our core explanatory variable, capturing the economic shock dimension, which is defined as the socioeconomic impact of the COVID-19 pandemic. The variable was an additive index composed of six individual indicators asking whether the girls' family had (i) lost employment or their source of livelihood, (ii) reduced work hours, (iii) faced financial shortages, (iv) gone hungry, (v) faced a higher care burden, and (vi) accommodated returning work migrants at home in consequence of the pandemic and its linked lockdown restrictions (see Table A1). Higher index scores thereby indicate greater economic pressure induced by the pandemic.

3.5 Analysis

Associations between explanatory variables on the individual, socio-economic, institutional, and external shock level with the likelihood of child marriage were estimated based on multivariable linear probability regression analyses that were run separately for the respective samples from India and Zambia.[7] For ease of interpretation, we also estimated a logistic regression model to calculate the predicted probability of child marriage for a low- and high-intensity economic shock scenario, with the latter being defined as a household having experienced at least two of the pandemic's economic repercussions listed in Table A1[8]. Analyses were carried out in R, Version 4.3.3. All data and code is available via https://osf.io/2xgpb/.

[7] We chose linear probability regression over logistic regression to facilitate the interpretation of coefficients, which indicate percentage-point changes in the likelihood of being exposed to child marriage for each included explanatory variable.

[8] The sampled age ranges are narrow (13–18 in India; 15–19 in Zambia), limiting the potential for meaningful non-linear effects. Within these age bands, the likelihood of child marriage tends to increase linear with age. Including an age-squared term would add unnecessary complexity.

3.6 Ethics

In India, the study was approved by the ethics committee of the Indian Institute of Technology Gandhinagar (IITG) in November 2021. In Zambia, the study was approved by the National Health Research Authority in July 2022. Both study components were additionally approved by the ethics committee of the medical faculty at the Technical University of Munich (TUM) on 29 December 2021.

4 Results

4.1 Socioeconomic Characteristics, Child Marriage, and Impact of the Pandemic

Descriptive information on girls' socio-demographic status, exposure to child marriage, and COVID-19 impacts are summarized in Table 1, separately for the sample from India and Zambia.

India

The average age of girls in the Indian sample was 15.6 years and more than two thirds of girls belonged to a lower caste. 16.5% of girls reported that they engaged in some form of income generation, and their households owned, on average, 6.7 assets. 28% of girls' mothers compared to 44% of girls' fathers had completed secondary school, thus reflecting stark gender disparities in parents' education status.

In our sample, 9% of adolescent girls were married underage. Brides' average age at marriage was 15.4 years, whereas the rate of being married increased with girls' age. Grooms were generally older than brides, with an average age of 22 years, thus also marking considerable age gaps between spouses. To illustrate this, more than 40% of the girls in our sample aged 18 years were already married (see Figure A1). The youngest bride in our sample was 13 years old when the wedding ceremony took place. Of the married girls, only 21% indicated that they got married because of love, whereas 71% indicated that their marriage was arranged by their family. Further, 9% of girls disclosed that they perceived their marriage as a forced marriage. A dowry was paid in 24% of the recorded marriages in the sample. We also captured girls' attitude towards child marriage by using a hypothetical scenario in which we asked them how they would feel if their sister or a close female friend were to get married before the age of 18. Notably, 88% of the girls in our sample disclosed that they would feel that *"she is too young to get married"* (see Table A2).

The economic impact that the COVID-19 pandemic on the families and households of girls in our

sample was substantial. Specifically, 60% of girls reported that at least one household member had lost their employment during the pandemic, and 55% reported productivity losses. In addition, 84% of households experienced financial shortages due to the pandemic and 35% of households struggled to provide enough foodfor everyone in the family. In 56% of households, the burden of unpaid work increased as a consequence of the pandemic, and in 10% of households, returning work migrants had to be accommodated as additional household members in the already strained family.

Zambia

The average age of girls in the Zambian sample was 16.7 years. 43% of these girls engaged in some form of income generation activity, and 30% lived in polygamous households, which could partly explain the generally bigger household size in the Zambian sample, relative to the Indian sample (7.8 household members versus 4.1 household members, respectively). Overall household wealth in Zambia was also lower than in India, with an average of 4.7 assets owned and living standard indicators achieved by the family. 34% of girls' mothers and 39% of girls' fathers had completed secondary education.

In Zambia, 7% of adolescent girls in our sample were married before they had reached age 18. The average marriage age was higher than in India, at 17.1 years (see Figure A2). The youngest bride in the Zambian sample was 15 years old. Of those married, 59% reported that they got married because of love - a larger share of married girls than in the Indian sample. Further reflecting this, only 3% of girls indicated that they were forced to get married by family members. In 66% of all reported marriages, a bride price was paid. Child marriage as a norm and practice was rejected more strongly among girls in Zambia as compared to girls in India. 98% of the Zambian girls in our sample claimed that they would feel that their sister or friend would be too young if she got married before reaching 18 years of age (see Table A2).

Lastly, the detrimental socioeconomic impacts of the COVID-109 pandemic were also reflected in the sample from Zambia, albeit less strongly than in India. Specifically, 6% of girls reported that at least one household member had lost their employment during the pandemic, and 12% reported that someone had faced productivity and income losses. 84% of households faced financial shortages caused by the pandemic, and 19% of respondents reported that they or someone in their family had to go without eating for a whole day because of lack of money and resources during the pandemic. The unpaid care burden increased in 13% of households, and 11% took in work migrants who came back to their rural villages during the pandemic.

Table 3: Sociodemographic and COVID-19 Impact Data from India and Zambia

	India		Zambia	
**Variable**	**Mean (SD)**	**n**	**Mean (SD)**	**n**
Sociodemographic Information				
Age	15.558 (1.482)	3049	16.716 (1.415)	1615
Lower /Scheduled Caste	0.669 (0.471)	3049		
Income generating activity	0.165 (0.371)	3049	0.043 (0.205)	1615
Number of assets/living standards indicators	6.700 (2.044)	3049	4.661 (2.111)	1615
Number of household members	4.082 (1.602)	3049	7.780 (4.146)	1651
Polygamous household			0.291 (0.454)	1615
Mother secondary education	0.284 (0.451)	3049	0.343 (0.475)	1615
Father secondary education	0.442 (0.496)	3049	0.387 (0.487)	1615
Child Marriage Indicators				
Child Marriage	0.091 (0.288)	3049	0.059 (0.236)	1615
Age at marriage bride	15.411 (1.876)	286	17.068 (1.039)	119
Age at marriage husband	22.035 (3.228)	286	20.687 (4.018)	119
Dowry/bride price paid	0.238 (0.426)	286	0.658 (0.476)	119
Love marriage	0.207 (0.405)	286	0.589 (0.493)	119
Arranged marriage (by parents)	0.705 (0.456)	286	0.376 (0.486)	119
Forced marriage	0.087 (0.283)	286	0.034 (0.182)	119
COVID-19 Impact				
Loss of employment during COVID	0.595 (0.490)	3094	0.064 (0.244)	1615
Reduced hours for income generation during COVID	0.551 (0.496)	3094	0.124 (0.330)	1615
Food shortage during COVID	0.351 (0.477)	3094	0.190 (0.392)	1615
More unpaid care work during COVID	0.559 (0.496)	3094	0.126 (0.332)	1615
Returning migrants in home during COVID	0.101 (0.301)	3094	0.111 (0.315)	1615
Financial shortages during COVID	0.841 (0.365)	3094	0.390 (0.487)	1615

Notes: Age at marriage, type of marriage, and financial transfers at marriage are only reported for the subsamples of married girls, namely 286 girls in India and 119 girls in Zambia.

4.2 Correlates of Child Marriage

We explored possible factors associated with child marriage on the (i) individual, (ii) socio-economic, (iii) institutional, and (iv) economic shock level based on separate linear probability regression models (see Figure 2).

In the sample from Zambia, only two factors were significantly associated with the outcome variable. Specifically, the strongest predictor of girls' marital status was their age, with a six percentage

Figure 2: Correlates of Child Marriage on Different Levels

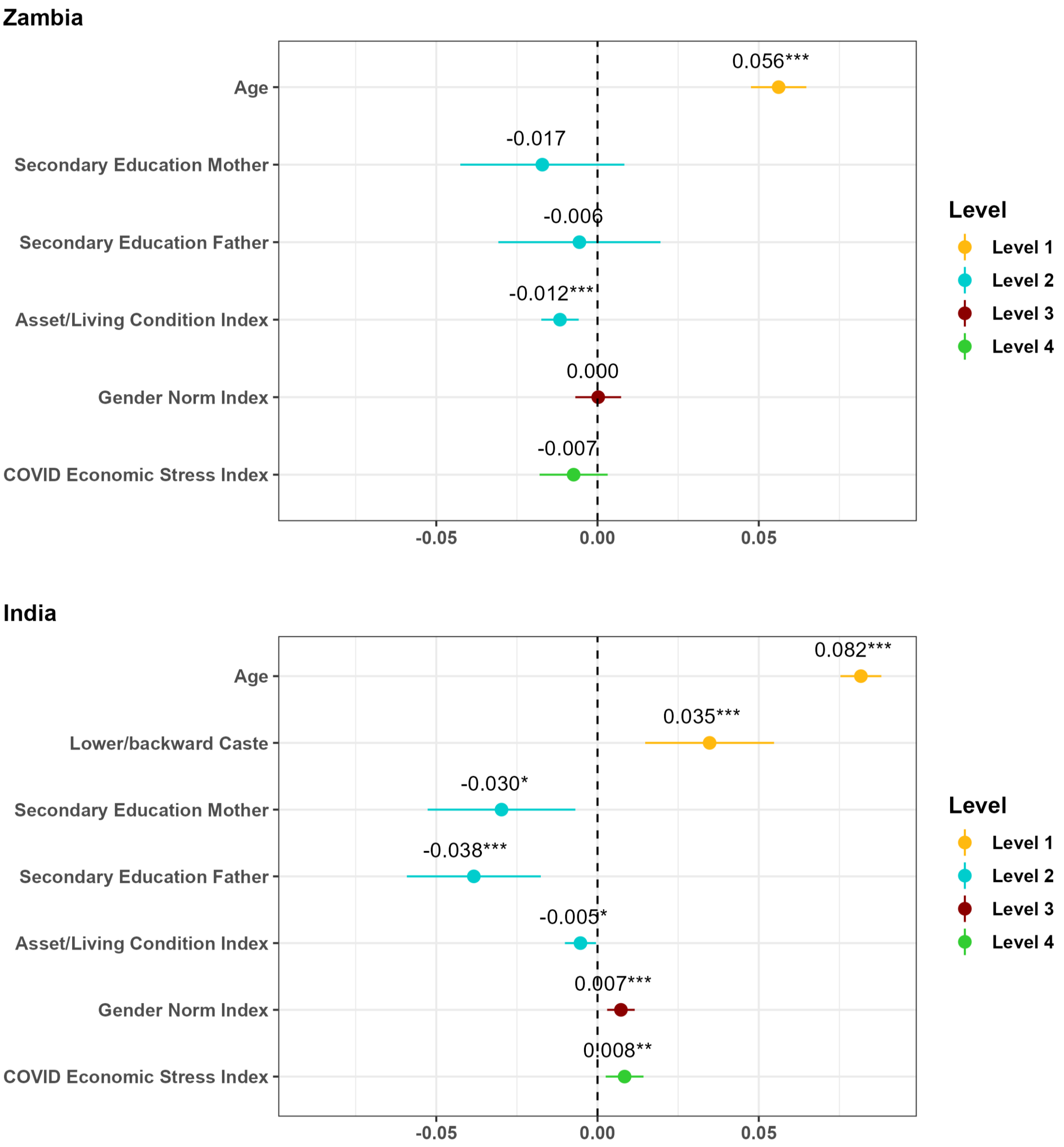

*Notes: Coefficients from linear probability regression model, *p<0.05, **p<0.01, ***p<0.001.*
Level 1 (yellow): individual level, Level 2 (blue): socio-economic level, Level 3 (red): institutional level, and Level 4 (green): economic shock level. Coefficients at different levels of the theoretical framework were estimated in separate models to avoid multicollinearity and endogeneity concerns. In each model, we controlled for individual-level factors, namely age and caste in India.

point increase in the probability of being married with every additional year of life. In addition, the rate of child marriages was significantly lower in households with higher living standards and wealth. The effect translates into a significant one percentage point decrease in the probability that a girl is married under age with every additional asset or living standard indicator that a household acquires. Notably, we did not observe any significant associations between child marriage and parents' education status or girls' internalized gender norms. Importantly, contrary to the predictions made by our *Hypothesis 1*, we do not observe a significant increase in child marriage rates among households that were more adversely affected by the economic pressures of the COVID-19 pandemic. In fact, the coefficient for the COVID Economic Stress Index even points in the opposite direction, albeit not being significant. Thus, we must reject *Hypothesis 1*, as we find no indication that the economic shock induced by the COVID-19 pandemic has prompted households to seek alternative income sources through a bride price and marrying off their daughters as a consumption-smoothing strategy.

In the sample from India, girls' age was also a significant correlate of child marriage, translating into an eight percentage point increase in girls' likelihood of being married with every year of age. Girls from lower castes were also more at risk of being married under age, whereby their probability of being married was 3.5 percentage points higher as compared to girls who were from higher castes. On the socio-economic level, girls whose fathers and/or mothers had completed secondary schooling were significantly less likely to become child brides, thus suggesting that parents' higher education is a protective factor with respect to child marriages. Similar to what we observed in Zambia, girls from economically better-off households were also less likely to be married under age, whereby any additional asset or living standard indicator translated into a 0.5 percentage-point decrease in the probability of child marriage. In addition, our analyses revealed a significant link between child marriage and the institutional-level factor of gender norms. Specifically, girls who were more approving of violence against women - reflecting stronger internalized patriarchal gender norms - were significantly more likely to be married before reaching the age 18.[9] Finally, our analysis fails to support the predictions made by our *Hypothesis 2*. While - based on our theoretical framework - we expected a decrease in child marriage rates within a dowry-based system like India, our regression reveals the opposite trend. Specifically, we observe a significant 0.8 percentage-point increase in the likelihood of girls being married underage with each additional economic pressure faced by a household due to the COVID-19 pandemic.

[9]The effect translates into a 0.7 percentage point increase in the probability of being married with every additional scenario (out of 7 scenarios) in which a girls thinks it is justifiable for a husband to beat his wife.

Lastly, for a more nuanced interpretation of the economic shock effect, we calculate the predicted probability of child marriage for a low- and high-intensity economic harm scenario. As shown in Figure 3, in Zambia, we do not observe any significant differences in the predicted probability of child marriage when comparing a hypothetical household that has experienced no substantial economic shock due to the pandemic with a hypothetical household that has suffered significant economic harm, for example through income losses or food shortages. Notably, the predicted probability even drops from 6.30 percent to 4.02 percent with higher economic pressures thus suggesting that families in Zambia were not resorting to child marriages with the intention to smooth consumption by means of a bride price. Conversely, in India, we report a significant increase in the predicted probability of child marriages: While in the low-intensity economic harm scenario, there is only a 6.50 percent chance that a girls is married under age, this risk almost doubles and increases to a 11.35 percent probability for girls to become child brides in the high-intensity economic harm scenario. The significant surge in the projected likelihood of child marriages in India implies that dowry payments did not function as a hindrance to the establishment of formal unions in face of the economic strains experienced during the COVID-19 pandemic.

Figure 3: Predicted Probability of Child Marriage for Different Economic Harm Scenarios

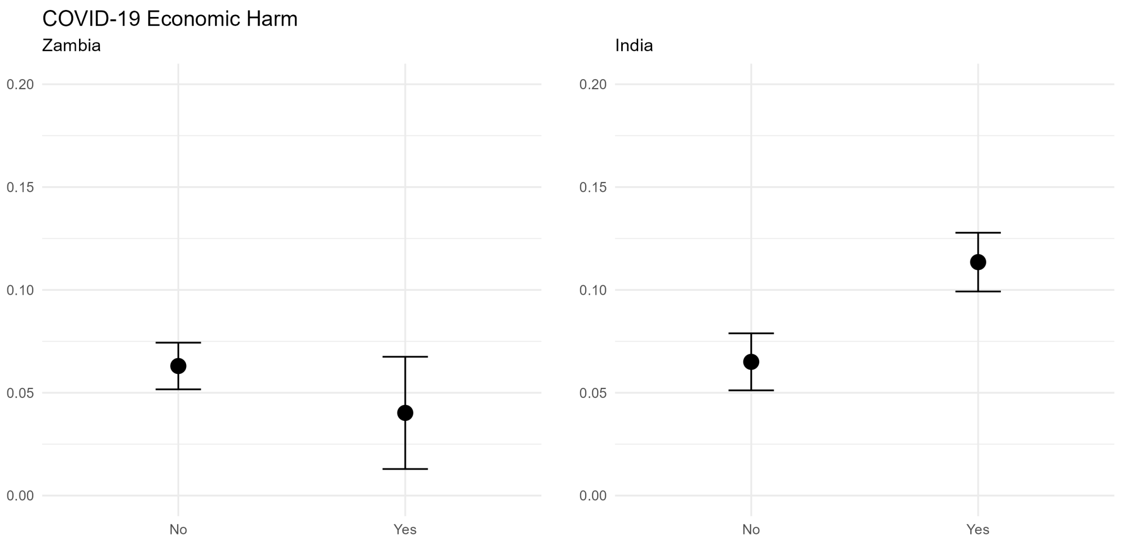

Notes: Predicted probabilities with robust standard errors based on logistic regression model, controlling for age and caste (in India). COVID-19 Economic Harm was coded as 1 ("yes") if a household had suffered two or more economic repercussions (see Table A1), and as 0 ("no") otherwise.

4.3 Qualitative findings on the associations between COVID-19 economic pressures, marriage customs, and child marriage

Zambia: Bride Price System

Our analysis in Zambia, a society that still holds bride price traditions, did not reveal an elevated risk of child marriage in the context of the COVID-19 pandemic, thereby refuting our *Hypothesis 1*. While our quantitative data could not confirm that the economic stress induced by the COVID-19 pandemic increased early marriage rates, the qualitative data from our focus group discussions can offer more nuanced insights into the potential factors driving still occurring child marriages during this period. Notably, one significant contributing factor might have been the school closures implemented due to the pandemic. These closures resulted in increased free time, allowing boys and girls to spend more time together and form relationships. As one adolescent girl described:

"Some days back, before Covid-19, we all [adolescent girls] used to go to school. There was no time to meet boys. But when Covid-19 started, schools started closing and that is when people had the chance to say 'ah! let us get married'. When schools closed, they [girls and boys] started marrying each other while they were still young."

Additionally, the prolonged closure of schools may have diminished girls' educational aspirations, shifting the trade-off between pursuing more years of schooling versus opting for early marriage in favor of the latter. Accordingly, a girl stated during the discussion:

"Even the thoughts of being in class had finished. They [friends from school] said they did not know what they were going to write, when they would go to class again, so they just thought of getting married. All the thoughts about school had vanished, there were only thoughts of playing that remained."

Lastly, the economic pressures induced by the pandemic may have made it more difficult for parents to afford their children's and particularly their daughters' school fees. This financial strain could have pushed these daughters into earlier marriages, as expressed in the following statement:

"Sometimes, what causes that [early marriage] is that there is no one to pay for her [adolescent girls'] school fee and she is just living at home. Then she will think of getting married instead of staying at home."

India: Dowry System

Our analysis of data from India, a society where dowry practices are prevalent, did not support *Hyothesis 2*. This hypothesis, aligned with Anderson (2007), proposed that child marriage rates would likely

decrease during economic shocks. A possible explanation for the increase in child marriages that we observe couls be that the groom's family did not demand dowry transfers, acknowledging the financial struggles many families faced during the pandemic. Indeed, only 24% of married girls indicated that a dowry was transferred for their marriage (see Table 1), and in 31% of the wedding ceremonies within our sample, there were reportedly no transfers of gifts such as jewellery, food, or gold. While alleviating financial distress for brides' families, these reduced dowry demands might also have made the pandemic period an opportune time for marrying off daughters without conventional economic obligations. Evidence gathered during focus group discussions and in-depth interviews corroborates this explanation, as captured in the following quotation by an adolescent girl from Sangli:

"During Corona, everyone faced financial losses and they [families] didn't have money for their daughter's wedding. So if they got a family who was not asking for much [dowry] then they married off the girl and there were no expenses towards the wedding as there would be no guests due to Corona [lockdown]."

Supporting this, a mother participating in a focus group discussion in Pune confirmed: *"The overall cost of the weddings has reduced a lot... really a lot."*. Similarly, a girls' father from Sangli noted: *"It [dowry] is still there, but it has reduced a lot. Recently, one of our relatives got married, the boy's family didn't take a single rupee."*

Another crucial factor appeared to be the costs associated with wedding ceremonies. In India, wedding ceremonies often involve several guests, and it is customary for the bride's family to bear this expense. However, our qualitative data highlighted that during the COVID-19 pandemic and the related contact restrictions, wedding ceremonies were significantly smaller, where only few guests were allowed to attend. Corroborating this, girls' parents confirmed during focus group discussions:

"Earlier [prior to the pandemic] there was the cost of guests, and also the cost for their invitations etc. Now hardly anyone is invited, and people also don't mind not being invited. They understand, that because of COVID, it is difficult to have regular weddings. Earlier, people would literally ask: "Why didn't you invite us for the wedding?" But now everyone knows and understands. Now even the children who are getting married, don't have such expectations; They're happy even if the ceremony takes place at home with immediate family."

Thus, the pandemic may have presented an attractive economic opportunity for parents to marry off their daughters at a significantly lower cost than usual. Some of the marriages even seemed to have been arranged hastily and in an ad-hoc fashion to benefit from the low-cost circumstance. Reflecting this, an adolescent girl from Pune described the following situation during an in-depth interview:

"Some people [adolescents in the participant's village] got married in the evening due to fear of police [during the lockdown] with very few people. They got married in the morning at 3 a.m. or 4 a.m. in the temple with only two three people present for the marriage. Even the girl did not know that she was going to get married."

Another mother added:

"Indian weddings always take place with great pomp. There are 2-3 hotels near where we live, and each time we see at least 300-400 cars parked, some distance from the hotels [for the ceremony]. So the expense is definitely extracted from the girls' family. But now [during the pandemic], a lot of the cost can be reduced, by having simple weddings in homes or in temples. My own cousins' brother got married recently, and he managed to save 5-10 lakhs[10] by having a very simple wedding, which we all attended virtually."

Lastly, by marrying off their daughter, the bride's natal family may benefit from having fewer mouths to feed, as she moves in with her in-laws, thereby reducing the strain on their limited resources. For instance, one girl recounted:

"For my marriage, my family did not have to carry any expenses as my father had told them my in-laws that he can only give the girl and nothing else since we are poor, but the boy's family had everything and took me.".

Similarly, some participants described how marrying off a daughter could help parents cope with the health-related pressures and expenses of the pandemic. Reflecting this, one father emphasized during a group discussion:*"If there is hospitalisation in the family, then it is good to get her [the daughter] married as it is difficult to continue her education then."*.

5 Discussion

Our study aimed to identify the effect of the COVID-19 pandemic and country-specific cultural factors on the risk of child marriage in India and Zambia.

In India, 9% of adolescent girls included in the study were married underage, with significant age gaps between spouses. Marriages were predominantly arranged by families, dowry was paid in 24% of the marriages and economic pressures during the pandemic increased the likelihood of child marriage.

Our findings on the individual, socio-economic, and institutional correlates of child marriages align with existing literature. Similar to previous studies, we also find that individual-level factors

[10] Between 6,000-11,000 EUR.

such as higher age and lower caste were associated with an increased risk of child marriage (Binu et al., 2022; Modak, 2019). In many LMICs, menarche translates into marriage eligibility, which is deeply rooted in patriarchal norms like the maintenance of purity before marriage and upholding the family reputation (Greene and Ellen, 2019; Liem et al., 2023; Fall, 2001). As a result, societal and familial pressure to marry off girls may intensify as they grow older. A previous study conducted with the same sample of 3049 adolescent girls in India presented that social expectations around marrying early were one of the main drivers of adolescent marriage and pregnancy (Shukla et al., 2023). Significant age differences between adolescent girls and their partners, as seen in this sample, can also lead to power imbalances in the relationship, leading to loss of sexual and reproductive health-related decision-making power and even intimate partner violence (Kidman, 2017).

The higher incidence of child marriage among lower caste families reveals the compounded disadvantages and social marginalisation faced by these groups. Other studies also corroborate these findings (Liczbińska et al., 2023; Paul, 2020). At the socio-economic level, parent's education level and household wealth were negatively associated with the probability of child marriage. Studies from other LMICs have also shown that higher parental education and wealth protect against child marriages (Bolarinwa et al., 2022; Muchomba, 2021; Pourtaheri et al., 2023). Additionally, the institutional-level factor of gender norms, here captured by a measure of justifying violence against women, emerged as a critical factor influencing the incidence of child marriage. Previous studies from India and Pakistan have also presented that women married as children were more likely to justify spousal violence (Nasrullah et al., 2017; Santhya et al., 2010). Others have highlighted that women who were married as children or married as adolescent girls face increased violence and controlling behaviour from their partners (Ahinkorah et al., 2023; Tenkorang, 2019; Steinert et al., 2023).

Finally, with regards to the impact of economic shocks, our analysis did not support the hypothesis that child marriage rates would likely decrease in consequence of the economic shock caused by the COVID-19 pandemic. In contrast, we found significantly higher rates of child marriages among families that experienced substantial economic hardships due to the pandemic. Nearly one-fifth of the child brides in our sample reported that the timing of their marriage was influenced by the pandemic, causing their marriages to occur earlier than they would have under normal circumstances. Qualitative interviews with married adolescents highlighted that the lack of dowry demand and low wedding costs made marrying during lockdown lucrative. While most previous studies observe a negative relationship between economic shocks and child marriage rates in dowry-based societies, a study from Bangladesh also presented that in the aftermath of a flood, marriages were less expensive

as most households suffered economically and social expectations around dowry and wedding expenses were lower, leading to families marrying off their daughters (Ahmed et al., 2019). Likewise, another study in the same setting from 2020 showed that households coped with economic hardship by marrying off their daughters to reduce household consumption (Carrico et al., 2020).

In Zambia, the rate of child marriage was slightly lower at 7%, with a higher proportion of marriages driven by romantic relationships rather than family arrangements. Higher household wealth was strongly associated with lower rates of child marriage in both countries. But while the COVID-19-related economic shock played a crucial role in the family's financial situation, it did not lead to an increase in child marriages when compared to our results from India.

Like in the Indian context, in Zambia as well, inequitable gender norms that prioritise women's roles as wives and household caretakers and men's role as the breadwinner often result in low investment in girls' education and future economic prospects. The drivers of child marriage in this sample were highlighted during interviews with girls. They mentioned that school closure due to the pandemic, economic pressures leading to non-payment of school fees, and prolonged periods of tedium due to the lockdown made families and girls lose interest in future education. Similar channels were also identified in a qualitative study in Indonesia after the pandemic (Rahiem, 2021). A combination of inequitable norms and a lack of avenues to pursue education pushes girls to form financially beneficial relationships in return for sex, leading to school dropout, adolescent pregnancies and subsequent child marriages (Fenn et al., 2015; Petroni et al., 2017; Steinhaus et al., 2016).

The significant association between household wealth and lower child marriage rates aligns with previous research from LMICs, which emphasises the protective effect of economic stability against early marriage (Hotchkiss et al., 2016; Rumble et al., 2018; Sayi and Sibanda, 2018; Yaya et al., 2019). In our sample, despite significant economic hardships that households faced during the COVID-19 pandemic, like loss of employment or income, there was no corresponding increase in child marriage rates, suggesting that Zambian families employed different coping mechanisms than their Indian counterparts. Families in Zambia did not marry their daughters in lieu of receiving the bride price as a consumption smoothing strategy. This finding is in contrast to previous studies that indicated an elevated risk of child marriages in societies with bride price traditions following natural disasters or income shocks (Kok et al., 2023; Anderson, 2007; Asadullah et al., 2021; Carrico et al., 2020; Das and Dasgupta, 2022; Dietrich et al., 2022; Khanna and Kochhar, 2021; Tsaneva, 2020). One possible explanation for our findings is that financial struggles during the pandemic affected all families, including

those of prospective grooms. This may have reduced their ability to afford a high and appealing bride price, leading parents of potential brides to be less motivated to marry off their daughters early during widespread economic hardship. This assumption is supported by married girls among whom less than 3% indicated that the primary reason for their marriage was economic aspects. In addition, the large majority of married girls (87%) reported that the timing of their wedding had not been affected by the pandemic.

This study has several limitations. The cross-sectional nature of the data does not allow us to draw causal conclusions on how the risk factors are linked to child marriage. To mitigate this, we present a conceptual framework based on previous studies and qualitative interviews with adolescent girls to unpack potential channels linking economic, social, cultural, and disruptive factors to child marriage outcomes. Apart from this, we did not collect any data prior to the pandemic based on which we could estimate the increase in child marriage rates caused by the pandemic. However, we apply an extensive list of measures that represent nuanced variations in the extent to which households were negatively affected by the pandemic. Lastly, data collection was limited to two rural and semi-urban locations in India and three rural locations in Zambia, which may affect the generalizability of our findings. Future studies should aim to collect longitudinal data and leverage quasi-experimental designs to establish causal relationships between the highlighted risk factors and child marriage. Additionally, incorporating qualitative interviews with not only adolescent girls but also their parents and relatives could provide deeper insights into the coping mechanisms and cultural contexts that drive child marriage, helping to unpack the nuanced ways in which economic, social, and cultural factors interact to influence marriage decisions.

Despite these shortcomings, our study has several key strengths. First, the comparative analysis between India and Zambia provides valuable cross-cultural insights, identifying common and unique factors influencing child marriage in different socio-economic and cultural contexts. Second, the use of a conceptual framework along with both quantitative and qualitative data offers a nuanced understanding of the drivers of child marriage, especially in relation to the COVID-19 pandemic. Lastly, this study includes a large sample of adolescent girls from diverse geographical locations within the two countries, enhancing the reliability of the findings.

The findings from this study have significant implications for intervention development and policy making aimed at reducing child marriage in LMICs both during public health emergencies (PHEs) and beyond. Restrictive gender norms are a significant barrier for girls in achieving their aspirations

and avoiding child marriage. Eroding this would require interventions to promote communication and support from their parents and community. Such programmes could help adolescents and parents to develop ways of coping with conflict and setting shared boundaries. One such intervention is the Families Matter! Program in Tanzania that aims to empower caregivers to discuss sexuality and practice positive parenting skills with their children (Kamala et al., 2017). Building on this, future interventions could be adapted to include discussions and activities specifically focused on sexual reproductive health and child marriage.

A recent systematic review of the association between public health emergencies (PHEs) and various gender outcomes highlighted that PHE-related response strategies like home confinement and closure of schools, along with associated economic losses at both national and household levels are key channels behind the increase in child marriage in LMICs (Shukla et al., 2023). While these measures are essential to curb new infections, governments and policymakers should focus on complementary measures to safeguard adolescents' health and life outcomes. These include providing safe spaces for them to socialise and receive education, organising mobile youth-friendly health and counselling services, subsidising or removing school fees to allow girls to return to school, and integrating child marriage prevention messages into the broader policy landscape (Bandiera et al., 2020; Dona et al., 2016; Mahato et al., 2015; Tirivayi et al., 2020; Rafaeli, 2020). Lastly, addressing structural barriers like poverty, low education, and limited employment opportunities requires continuous development of policies and programmes at a national level to increase the economic resilience of families, especially during crises. This could include social protection programs, job creation initiatives, and support for small businesses.

References

Abdurahman, D., N. Assefa, and Y. Berhane (2023): "Adolescent Girls' Early Marriage Intention and its Determinants in Eastern Ethiopia: A Social Norms Perspective," *Sage Open*, 13.

Ahinkorah, B. O., R. G. Aboagye, J. Okyere, A.-A. Seidu, E. Budu, and S. Yaya (2023): "Child marriage and its association with partner controlling behaviour against adolescent girls and young women in sub-Saharan Africa," *BMC Global and Public Health*, 1, 9.

Ahmed, K. J., S. M. A. Haq, and F. Bartiaux (2019): "The nexus between extreme weather events, sexual violence, and early marriage: a study of vulnerable populations in Bangladesh," *Population and Environment*, 40, 303–324.

Ahmed, R. (2018): "The impact of flooding on marriage: evidence from Pakistan," *Disaster Prevention and Management*, 27, 115–128.

Ahonsi, B., K. Fuseini, D. Nai, E. Goldson, S. Owusu, I. Ndifuna, I. Humes, and P. L. Tapsoba (2019): "Child marriage in Ghana: evidence from a multi-method study," *BMC Women's Health*, 19, 126.

Akter, S., C. Williams, A. Talukder, M. N. Islam, J. V. Escallon, T. Sultana, N. Kapil, and M. Sarker (2021): "Harmful practices prevail despite legal knowledge: a mixed-method study on the paradox of child marriage in Bangladesh," *Sexual and Reproductive Health Matters*, 29, 1885790.

Al-Ridhwany, H. H. and A. A. Al-Jawadi (2014): "Early Child Marriage in Mosul at North of Iraq : Prevalence and Preference," *World Family Medicine Journal/Middle East Journal of Family Medicine*, 12, 9–16.

Alem, A. Z., Y. Yeshaw, S. A. Kebede, A. M. Liyew, G. A. Tesema, C. D. Agegnehu, and A. B. Teshale (2020): "Spatial Distribution and Determinants of Early Marriage among Married Women in Ethiopia: A spatial and Multilevel Analysis," *BMC Women's Health*, 20, 207.

Ali, A., I. Ibrahim, S. Abdelgbar, and M. Elgessim (2014): "Socio-demographic factors affecting child marriage in Sudan," *Journal of Women´s Health Care*, 3, 2167–0420.

Alston, M., K. Whittenbury, A. Haynes, and N. Godden (2014): "Are climate challenges reinforcing child and forced marriage and dowry as adaptation strategies in the context of Bangladesh?" *Women's Studies International Forum*, 47, 137–144.

Anderson, S. (2007): "The economics of dowry and brideprice," *Journal of Economic Perspectives*, 21, 151–174.

Asadullah, M. N., K. M. M. Islam, and Z. Wahhaj (2021): "Child marriage, climate vulnerability and natural disasters in coastal Bangladesh," *Journal of Biosocial Science*, 53, 948–967.

Aychiluhm, S. B., A. K. Tesema, and A. W. Tadesse (2021): "Early Marriage and Its Determinants among Married Reproductive Age Group Women in Amhara Regional State, Ethiopia: A Multilevel Analysis," *BioMed Research International*, 2021, 1969721.

Bandiera, O., N. Buehren, M. Goldstein, I. Rasul, and A. Smurra (2020): "Do school closures during an epidemic have persistent effects? Evidence from Sierra Leone in the time of Ebola," *J-PAL Working Paper*, https://www.povertyactionlab.org/sites/default/files/research-paper/working-paper_720_School-Closures-During-Epidemic_Sierra-Leone_July2020.pdf, Accessed 4 April 2024.

Batheja, D., A. Nandi, A. Banerji, A. Summan, and R. Laxminarayan (2022): "Covid-19 and Women's Age at Marriage: New Evidence from India," *SSRN Electronic Journal*.

Binu, V., V. Sridhar, S. Subba, P. Prathyusha, and K. Sabu (2022): "Direct and indirect factors associated with child marriage: Evidence from India using NFHS-4 data," *Child Abuse & Neglect*, 131, 105785.

Blanco, L. (2023): "Shifting Marriage Timing for Women: Natural disasters and Forced displacement," *Unpuplished manuscript, Available:*, http://congress-files.s3.amazonaws.com/2022-08/mu%c3%b1oz-blanco_laura_women%27s_marriage_and_natural_disasters.pdf, Accessed: 6 May 2024.

Bloch, F. and V. Rao (2002): "Terror as a Bargaining Instrument: A Case Study of Dowry Violence in Rural India," *American Economic Review*, 92, 1029–1043.

Bolarinwa, O. A., B. O. Ahinkorah, J. Okyere, A.-A. Seidu, and O. S. Olagunju (2022): "A multilevel analysis of prevalence and factors associated with female child marriage in Nigeria using the 2018 Nigeria Demographic and Health Survey data," *BMC Women's Health*, 22, 158.

Bottan, N., B. Hoffmann, and D. Vera-Cossio (2020): "The unequal impact of the coronavirus pandemic: Evidence from seventeen developing countries," *Plos One*, 15, e0239797.

Brandl, E. and H. Colleran (2024): "Does bride price harm women? Using ethnography to think about causality," *Evolutionary Human Sciences*, 6, e29.

Buchmann, N., E. Field, R. Glennerster, S. Nazneen, and X. Y. Wang (2023): "A Signal to End Child Marriage: Theory and Experimental Evidence from Bangladesh," *American Economic Review*, 113, 2645–2688.

Buheji, M., K. Da Costa Cunha, G. Beka, B. Mavrić, Y. Leandro do Carmo de Souza, S. Da Souza Costa Silva, M. Hanafi, and T. Chetia Yein (2020): "The Extent of COVID-19 Pandemic Socio-Economic Impact on Global Poverty. A Global Integrative Multidisciplinary Review," *American Journal of Economics*, 10, 213–224.

Bundervoet, T., M. E. Dávalos, and N. Garcia (2022): "The short-term impacts of COVID-19 on households in developing countries: An overview based on a harmonized dataset of high-frequency surveys," *World Development*, 153, 105844.

Caldwell, J. C., P. Caldwell, B. K. Caldwell, and I. Pieris (1998): "The Construction of Adolescence in a Changing World: Implications for Sexuality, Reproduction, and Marriage," *Studies in Family Planning*, 29, 137.

Carrico, A. R., K. M. Donato, K. B. Best, and J. Gilligan (2020): "Extreme weather and marriage among girls and women in Bangladesh," *Global Environmental Change*, 65, 102160.

Child Marriage Data Portal (2025): "Global Trends & SDG Progress," https://childmarriagedata.org/, Accessed: 22 May 2025.

Chort, I., R. Hotte, and K. Marazyan (2025): "Income Shocks, Bride Price, and Child Marriage in Turkey," *The World Bank Economic Review*.

Cislaghi, B., G. Mackie, P. Nkwi, and H. Shakya (2019): "Social norms and child marriage in Cameroon: An application of the theory of normative spectrum," *Global Public Health*, 14, 1479–1494.

Cislaghi, B., P. Nkwi, G. Mackie, and H. Shakya (2020): "Why context matters for social norms interventions: The case of child marriage in Cameroon," *Global Public Health*, 15, 532–543.

Collin, M. and T. Talbot (2017): *Do Age-of-Marriage Laws Work? Evidence from a Large Sample of Developing Countries*, https://papers.ssrn.com/sol3/papers.cfm?abstract_id=3013388, Accessed: 3 July 2024.

Corno, L., N. Hildebrandt, and A. Voena (2020): "Age of Marriage, Weather Shocks, and the Direction of Marriage Payments," *Econometrica*, 88, 879–915.

Corno, L. and A. Voena (2023): "Child marriage as informal insurance: Empirical evidence and policy simulations," *Journal of Development Economics*, 162, 103047.

Cousins, S. (2020): "2·5 million more child marriages due to COVID-19 pandemic," *The Lancet*, 396, 1059.

Das, S. and S. Dasgupta (2022): "Marriage market responses in the wake of a natural disaster in India," *Available at SSRN 3266117*.

Delprato, M., K. Akyeampong, and M. Dunne (2017): "Intergenerational education effects of early marriage in sub-Saharan Africa," *World Development*, 91, 173–192.

Dharan, N. and R. B. Sah (2014): "Factors affecting early age marriage in Morang District of Nepal," *Ethnicity*, 293, 0–342.

Dietrich, S., A. Meysonnat, F. Rosales, V. Cebotari, and F. Gassmann (2022): "Economic development, weather shocks and child marriage in South Asia: A machine learning approach," *Plos One*, 17, 0271373.

Diop, S. and S. A. Asongu (2023): "The Covid-19 Pandemic and the New Poor in Africa: The Straw That Broke the Camel's Back," *Forum for Social Economics*, 52, 143–154.

Dona, R., A. Alano, and R. Villas (2016): "Adolescent Girls in Disaster & Conflict: Interventions for Improving Access to Sexual and Reproductive Health Services," *United Nations Population Fund*, report, https://www.unfpa.org/sites/default/files/pub-pdf/UNFPA-Adolescent_Girls_in_Disaster_Conflict-Web.pdf,
Accessed 12 June 2024.

Envuladu, E. A., R. J. Umaru, N. O. Iorapuu, I. A. Osagie, E. O. Okoh, and A. L. Zoakah (2016): "Determinants and effect of girl child marriage: a cross sectional study of school girls in Plateau State, Nigeria," *International Journal of Medicine and Biomedical Research*, 5, 122–129.

Equality Now (2020): "How COVID-19 is impacting efforts to end child marriage across Africa," https://equalitynow.org/news_and_insights/covid_19_child_marriage_africa/, Accessed: 20 May 2025.

Equality now (2024): "Illegal to marry below 18 - Zambia passes the landmark," https://equalitynow.org/press_release/illegal-to-marry-below-18-zambia-passes-the-landmark\-marriage-amendment-act-2023/, Accessed 1 August 2024.

Fall, S. (2001): "Menarche to Marriage: Sexual Health Behaviors of Young Women in Rural Gujarat, India," UCHC Graduate School Masters Theses 2003 - 2010. 40, Available: https://digitalcommons.lib.uconn.edu/uchcgs_masters/40/, Accessed 7 August 2024.

Fattah, K. N. and S. Camellia (2022): "Poverty, dowry and the 'good match': revisiting community perceptions and practices of child marriage in a rural setting in Bangladesh," *Journal of Biosocial Science*, 54, 39–53.

Fenn, N., J. Edmeades, H. Lantos, and O. Onovo (2015): "Child marriage, Adolescent pregnancy and Family formation in West and Central Africa," Report: Available https://www.unicef.org/wca/reports/child-marriage-adolescent-pregnancy-and-family-formation-west-and-central-africa, Accessed 10 March 2024.

Field, E. and A. Ambrus (2008): "Early Marriage, Age of Menarche, and Female Schooling Attainment in Bangladesh," *Journal of Political Economy*, 116, 881–930.

Girls not brides (2024): "The impact of the law on child marriage and girls' rights," Report: Available https://www.girlsnotbrides.org/learning-resources/resource-centre/impact-law-child-marriage-girls-rights/, Accessed 30 July 2024.

Godha, D., D. R. Hotchkiss, and A. J. Gage (2013): "Association between child marriage and reproductive health outcomes and service utilization: a multi-country study from South Asia," *The Journal of Adolescent Health*, 52, 552–558.

Government India (2006): "The prohibition of child marriage," https://www.indiacode.nic.in/bitstream/123456789/15943/1/the_prohibition_of_child_marriage_act%2C_2006.pdf, Accessed 3 August 2024.

Greene, M. and S. Ellen (2019): "Social and gender norms and child marriage," *Advanced Learning and Information on Gender Norms*.

Haq, S., A. Mufti, L. Shamin, and A. Arnika (2024): "Early marriage of girls in the context of the COVID-19 pandemic: a literature review," *Humanities and Social Sciences Communications*, 11, 1–11.

Hassfurter, K. (2021): "COVID-19: A threat to progress against child marriage," *UNICEF*, https://data.unicef.org/resources/covid-19-a-threat-to-progress-against-child-marriage/, Accessed 1 Aril 2024.

Hofisi, T. M. and C. Hungwe (2025): ""One thing led to another" - teenage pregnancy during COVID-19: a Zimbabwean case study," *International Journal of Adolescence and Youth*, 30.

Hoogeveen, J., B. Van Der Klaauw, and G. Van Lomwel (2011): "On the timing of marriage, cattle, and shocks," *Economic Development and Cultural Change*, 60, 121–154.

Hossain, M. J., M. A. Soma, M. S. Bari, T. B. Emran, and M. R. Islam (2021): "COVID-19 and child marriage in Bangladesh: emergency call to action," *BMJ Paediatrics Open*, 5, e001328.

Hotchkiss, D. R., D. Godha, A. J. Gage, and C. Cappa (2016): "Risk factors associated with the practice of child marriage among Roma girls in Serbia," *BMC International Health and Human Rights*, 16, 6.

India Child Protection (2025): "India Child Protection," https://www.childprotection.org.in/end-child-marriage, Accessed: 22 May 2025.

Jibon, M. J. N., M. R. S. Ritu, M. M. Islam, S. Islam, M. A. Rahman, and M. A. Rakib (2025): "Combined Impact of Climate Change, Local Hazards, and COVID-19 Increases Child Marriage Risks in Char Land Communities of Northern Bangladesh," *Global Social Welfare*, 1–12.

Julianto, V., K. Kamsi, A. Haq, R. L. Shofa, and A. Liem (2025): "Tick tick boom: the rise of child marriage in Indonesia during the COVID-19 pandemic," *Vulnerable Children and Youth Studies*, 20, 131–147.

Kamala, B. A., K. D. Rosecrans, T. A. Shoo, H. Z. Al-Alawy, F. Berrier, D. F. Bwogi, and K. S. Miller (2017): "Evaluation of the families matter! Program in Tanzania: An intervention to promote effective parent-child communication about sex, sexuality, and sexual risk reduction," *AIDS Education and Prevention*, 29.

Khan, S. (2024): "Female education and marriage in Pakistan: The role of financial shocks and marital customs," *World Development*, 173, 106413.

Khanna, M. and N. Kochhar (2021): "Do marriage markets respond to a natural disaster? The impact of flooding of the Kosi river in India," *Journal of Population Economics*, 36, 2241–2276.

Kidman, R. (2017): "Child marriage and intimate partner violence: a comparative study of 34 countries," *International Journal of Epidemiology*, 46, 662–675.

Kohno, A., T. Techasrivichien, S. P. Suguimoto, M. Dahlui, N. D. Nik Farid, and T. Nakayama (2020): "Investigation of the key factors that influence the girls to enter into child marriage: A meta-synthesis of qualitative evidence," *Plos One*, 15, e0235959.

Kok, M. C., T. Kakal, A. B. Kassegne, I. M. Hidayana, A. Munthali, J. A. Menon, P. Pires, T. Gitau, and A. van der Kwaak (2023): "Drivers of child marriage in specific settings of Ethiopia, Indonesia, Kenya, Malawi, Mozambique and Zambia - findings from the Yes I Do! baseline study," *BMC Public Health*, 23, 794.

Liczbińska, G., M. Brabec, R. K. Gautam, J. Jhariya, and A. Kumar (2023): "From little girls to adult women: Changes in age at marriage in Scheduled Castes from Madhya Pradesh and Uttar Pradesh, India," *Plos One*, 18, e0281506.

Liem, A., V. Julianto, Kamsi, A. Haq, R. L. Shofa, and Kamilatissa'adah (2023): "Factors associated with child marriage during the COVID-19 pandemic in Indonesia," *Contemporary Islam*, 1–22.

Mahato, P. K., P. R. Regmi, E. van Teijlingen, P. Simkhada, C. Angell, and B. Sathian (2015): "Birthing centre infrastructure in Nepal post 2015 earthquake," *Nepal Journal of Epidemiology*, 5, 518.

Makino, M., A. S. Shonchoy, and Z. Wahhaj (2023): "Early effects of the COVID-19 lockdown on children in north-western Bangladesh," *Oxford Development Studies*, 1–20.

Melnikas, A. J., G. Saul, N. Pandey, M. Makino, S. Amin, and M. Chau (2023): "Do Child Marriage Programs Help Girls Weather Shocks Like COVID-19? Evidence from the More Than Brides Alliance Intervention*," *Economic Papers: A Journal of Applied Economics and Policy*, 42, 395–407.

Menon, S. (2020): "The effect of marital endowments on domestic violence in India," *Journal of Development Economics*, 143, 102389.

Miguel, E. and A. M. Mobarak (2022): "The Economics of the COVID-19 Pandemic in Poor Countries," *Annual Review of Economics*, 14, 253–285.

Modak, P. (2019): "Determinants of girl-child marriage in high prevalence states in India," *Journal of International Women's Studies*, 20, 374–394.

Muchomba, F. M. (2021): "Parents' assets and child marriage: Are mother's assets more protective than father's assets?" *World Development*, 138, 105226.

Musa, S. S., G. O. Odey, M. K. Musa, S. M. Alhaj, B. A. Sunday, S. M. Muhammad, and D. E. Lucero-Prisno (2021): "Early marriage and teenage pregnancy: The unspoken consequences of COVID-19 pandemic in Nigeria," *Public Health in Practice*, 2, 100152.

Nasrullah, M., S. Muazzam, F. Khosa, and M. M. H. Khan (2017): "Child marriage and women's attitude towards wife beating in a nationally representative sample of currently married adolescent and young women in Pakistan," *International Health*, 9, 20–28.

NITI Aayog (2021): "India national Multidimensional poverty index Baseline report based on NFHS-4 (2015-16)," Report, Availabe https://ophi.org.uk/publications/India-mpi-report-2021, Accessed 4 August 2024.

Pacheco-Montoya, D., E. Murphy-Graham, E. E. V. López, and A. K. Cohen (2022): "Gender norms, control over girls' sexuality, and child marriage: A Honduran case study," *Journal of Adolescent Health*, 70, S22–S27.

Paul, P. (2020): "Child Marriage Among Girls in India: Prevalence, Trends and Socio-Economic Correlates," *Indian Journal of Human Development*, 14, 304–319.

Petroni, S., M. Steinhaus, N. S. Fenn, K. Stoebenau, and A. Gregowski (2017): "New findings on child marriage in sub-Saharan Africa," *Annals of Global Health*, 83.

Population Council, UNFPA (2024): "Child Marriage Research in Zambia," https://zambia.unfpa.org/sites/default/files/pub-pdf/Child%20Marriage%20in%20Zambia.pdf],Accessed31July2024, Accessed 10 Mai 2024.

Pourtaheri, A., S. B. T. Sany, M. A. Aghaee, H. Ahangari, and N. Peyman (2023): "Prevalence and factors associated with child marriage, a systematic review," *BMC Women's Health*, 23, 531.

Psaki, S. R., A. J. Melnikas, E. Haque, G. Saul, C. Misunas, S. K. Patel, T. Ngo, and S. Amin (2021): "What Are the Drivers of Child Marriage? A Conceptual Framework to Guide Policies and Programs," *The Journal of Adolescent Health*, 69, S13–S22.

Rafaeli, T. (2020): "The links between girls' life skills intervention in emergencies and their return to education post-crisis and prevention of unwanted pregnancies and early marriage," *Knowledge, Evidence and Learning for Development*.

Rahiem, M. D. H. (2021): "COVID-19 and the surge of child marriages: A phenomenon in Nusa Tenggara Barat, Indonesia," *Child Abuse & Neglect*, 118, 105168.

Raj, A., N. Saggurti, D. Balaiah, and J. G. Silverman (2009): "Prevalence of child marriage and its effect on fertility and fertility-control outcomes of young women in India: a cross-sectional, observational study," *The Lancet*, 373, 1883–1889.

Rumble, L., A. Peterman, N. Irdiana, M. Triyana, and E. Minnick (2018): "An empirical exploration of female child marriage determinants in Indonesia," *BMC Public Health*, 18, 407.

Santhya, K. G., U. Ram, R. Acharya, S. J. Jejeebhoy, F. Ram, and A. Singh (2010): "Associations between early marriage and young women's marital and reproductive health outcomes: evidence from India," *International Perspectives on Sexual and Reproductive Health*, 132–139.

Sayi, T. S. and A. Sibanda (2018): "Correlates of Child Marriage in Zimbabwe," *Journal of Family Issues*, 39, 2366–2388.

Shukla, S., J. A. Ezebuihe, and J. I. Steinert (2023): "Association between public health emergencies and sexual and reproductive health, gender-based violence, and early marriage among adolescent girls: a rapid review," *BMC Public Health*, 23, 117.

Steinert, J. I., H. Prince, J. Ezebuihe, and S. Shukla (2023): "Violence Against Adolescent Girls During the COVID-19 Pandemic: Quantitative Evidence From Rural and Urban Communities in Maharashtra, India," *The Journal of Adolescent Health*, 73, 1010–1018.

Steinhaus, M., L. Hinson, A. T. Rizzo, and A. Gregowski (2019): "Measuring Social Norms Related to Child Marriage Among Adult Decision-Makers of Young Girls in Phalombe and Thyolo, Malawi," *The Journal of Adolescent Health*, 64, S37–S44.

Steinhaus, M., N. Stevanovic Fenn, S. Petroni, and A. Gregowski (2016): "She cannot just sit around waiting to turn twenty," *International Center for Research on Women*, report, Available https://www.icrw.org/publications/she-cannot-just-sit-around-waiting-to-turn-twenty/, Accessed 10 July 2024.

Tenkorang, E. Y. (2019): "Explaining the links between child marriage and intimate partner violence: Evidence from Ghana," *Child Abuse & Neglect*, 89, 48–57.

Tewahido, D., A. Worku, A. W. Tadesse, H. Gulema, and Y. Berhane (2022): "Adolescent girls trapped in early marriage social norm in rural Ethiopia: A vignette-based qualitative exploration," *Plos One*, 17, e0263987.

Tirivayi, N., D. Richardson, M. Gavrilovic, V. Groppo, L. Kajula, E. Valli, and F. Viola (2020): "A rapid review of economic policy and social protection responses to health and economic crises and their effects on children: Lessons for the COVID-19 pandemic response," *United Nations*, Report https://www.un-ilibrary.org/content/papers/25206796/174, Accessed 10 April 2024.

Trinh, T.-A. and Q. Zhang (2021): "Adverse shocks, household expenditure and child marriage: evidence from India and Vietnam," *Empirical Economics*, 61, 1617–1639.

Tsaneva, M. (2020): "The Effect of Weather Variability on Child Marriage in Bangladesh," *Journal of International Development*, 32, 1346–1359.

UNICEF (2024): "Child marriage," https://www.unicef.org/protection/child-marriage, Accessed 4 January 2024.

United Nations (2024): "United Nations: Gender equality and women's empowerment," https://www.un.org/sustainabledevelopment/gender-equality/, Accessed 20 October 2023.

United Nations Children´s Fund (2021): "Towards Ending Child Marriage: Global trends and profiles of progres," https://data.unicef.org/resources/towards-ending-child-marriage/, Accessed 2 February 2024.

United Nations Population Fund (2020): "Child Marriage and the Law: Technical Note for the Global Programme to End Child Marriage," https://www.unfpa.org/resources/child-marriage-and-law-technical-note-global-programme-end-child-marriage, Accessed 23 July 2024.

Wilson, N. (2022): "Child Marriage Bans and Female Schooling and Labor Market Outcomes: Evidence from Natural Experiments in 17 Low- and Middle-Income Countries," *American Economic Journal: Economic Policy*, 14, 449–477.

World Bank (2018): "India States Briefs," *World Bank Group*, https://www.worldbank.org/en/news/feature/2016/05/26/india-states-briefs, Accessed 9 July 2024.

Yaya, S., E. K. Odusina, and G. Bishwajit (2019): "Prevalence of child marriage and its impact on fertility outcomes in 34 sub-Saharan African countries," *BMC International Health and Human Rights*, 19, 33.

Supplemenary Files

Table A1: Construction of Index Measures

Variable	Individual Indicators	Aggregation
Asset/Living Condition Index	*Which of the following does your household own:*<br>Car, Bike, Cycle, TV, Radio, Phone, Fridge, Goat,<br>Cattle, Pigs, Sheep, Flush, Tapwater, Electricity	Additive sum score indicating<br>number of indicators owned
Gender Norm Index	*In which situations should a husband be allowed*<br>*to beat his wife?*<br>If she...<br>... goes out without telling him<br>... argues with him<br>... refuses to have sex with him<br>... burns the food<br>... hides information from him<br>... hides money from him<br>... falls sick and there is a chance she may infect him	Additive sum score indicating number<br>of statements respondent agrees with
COVID Economic Stress Index	*Because of the pandemic, did you or*<br>*someone in your family...*<br>... lose their employment/source of livelihood?<br>... have to reduce their work hours?<br>... experience financial resource shortages?<br>... go without eating for a whole day because<br>of lack of money or resources?<br>... have to increase time spent doing household chores,<br>including taking care of sick people?<br>Do you have any household members who had migrated<br>for work and came home or tried<br>to return home?	Additive sum score indicating number<br>of economic stressors experienced during<br>COVID-19 pandemic and lockdown

Table A2: Girls' Attitudes Towards Child Marriage

**How would you feel if your friend or sister who is less than 18 years old got married?**			
Country	Response	Absolute value	Relative value
Zambia	Would be happy and celebrate	39	2.493%
	Would feel she is too young to get married	1525	97.507%
India	Would be happy and celebrate	339	11.629%
	Would feel she is too young to get married	2576	88.371%

Table A3: Economic and Temporal Aspects of Marriages

Country	Variable	Absolute value	Relative value
Zambia	My husband and I got married because the proposal was good in terms of economic aspects	3	2.56%
	The timing of my wedding was not affected by the pandemic	101	87.07%
	My wedding happened earlier because of the pandemic	7	6.03%
India	My husband and I got married because the proposal was good in terms of economic aspects	18	6.50%
	The timing of my wedding was not affected by the pandemic	203	73.29%
	My wedding happened earlier because of the pandemic	54	19.49%

Figure A1: Child Marriage Rate by Girls' Age: India

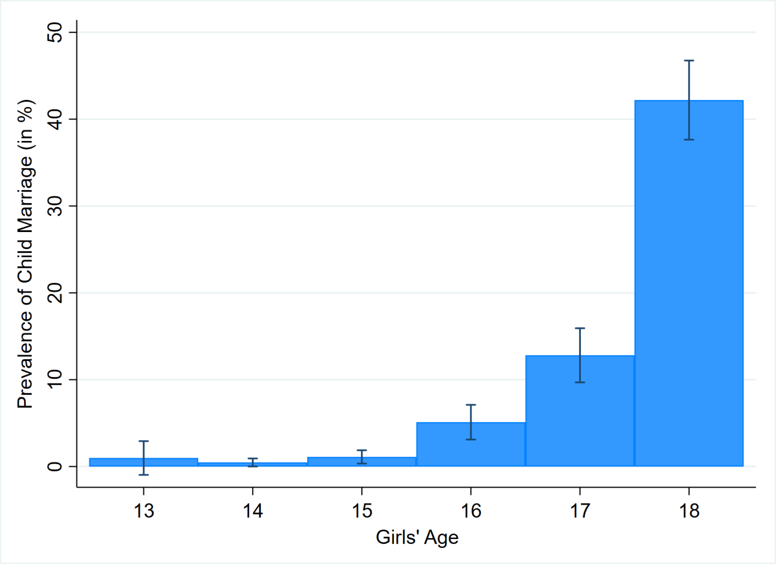

Figure A2: Child Marriage Rate by Girls' Age: Zambia

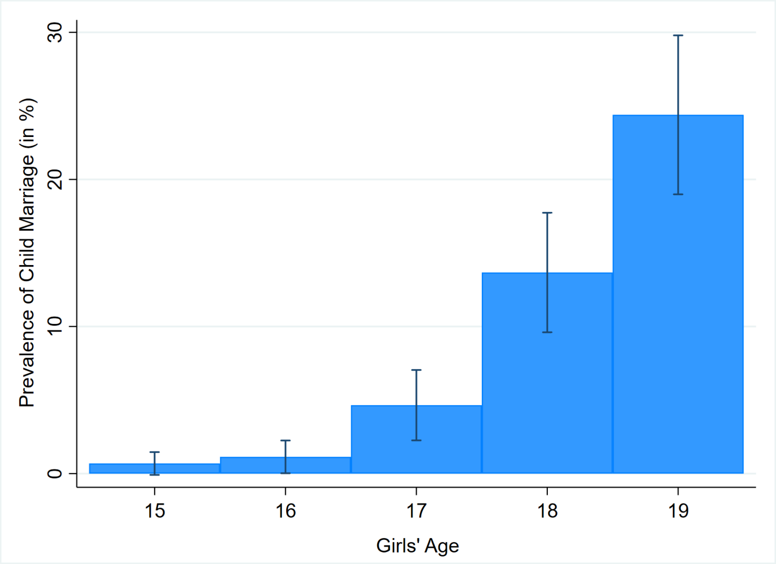

Click here to access/download
Supporting Information
IITGN_IEC_2025-26_23-Decision Letter.pdf

Ethics Approval Zambia

Click here to access/download
Supporting Information
NHRA Approval.pdf

Ethics Approval TUM English Translation

Click here to access/download

Supporting Information

TUM Ethics Translation.pdf
