# Supplementary material for: The interplay of economic shocks and cultural practices in child marriage: Comparative evidence from India and Zambia during the COVID-19 pandemic
Source: PLoS One. 2026 Jun 3;21(6):e0346851. doi: 10.1371/journal.pone.0346851 (PMC13232802; doi:10.1371/journal.pone.0346851)
Supplement: Attachment — Submitted filename: Response Letter_Child Marriage .pdf [file pone.0346851.s009.pdf]

Response Letter

Dear Reviewers,

We would like to thank you for the thorough assessment of our manuscript and the range of very helpful and insightful comments. We have now revised the paper to reflect your suggestions and believe that the manuscript has benefitted greatly from your input.

Below, we provide a detailed outline of all the edits that we have made. We reproduce each of your comments (in bold) and then elaborate on how we have addressed them (in blue). All edits are highlighted in the revised manuscript in track changes. Please note that all page numbers indicated here refer to the manuscript version where track changes are highlighted in red.

Reviewer 1

Thank you very much for reviewing our paper and for commending the quality of the writing and rigour of the methodology. Your feedback was crucial for making our paper more focused and in providing more context. Below, we provide detailed responses to your comments and questions.

1. **The authors have used a different author references that is used in PLOS ONE (i.e., numbering each citation rather than adding the authors in parenthesis). So, they may need to change this format in their manuscript.**

Thank you for the careful review of our manuscript and for flagging this important point. We have now adjusted the citation style to Vancouver, thus adhering to the PLOS ONE author guidelines throughout.

2. **One can see that the manuscript was developed very close to the pandemic. But if it is published, given that it is several years AFTER the pandemic, the descriptions should be in the past time.**

Thank you very much for noting this and we agree. We have carefully revised the write-up and have now ensured that any reference to the pandemic as well as the description of the study procedures is written in past tense.

3. **This is the place to use dowry, after having defined it.**

Thank you for this valuable suggestion. We agree that introducing the term at this point improves readability and helps readers understand its later use in the literature review. We have therefore decided to rewrite the paragraph. The new version is copied below:

"Further, marriage decisions can be driven by economic considerations, particularly when families face financial shortages and economic pressure. In patrilocal contexts, where it is customary for the bride to move in with her family-in-law after marriage, marrying off a daughter can reduce the economic burden on her natal household, as scarce financial resources and food no longer need to be shared with one additional household member. In many such settings, this incentive may coexist with dowry institutions, whereby the bride's family transfers assets or payments to the groom's family at marriage (17; 18). Hence, in such systems, the economic benefits of reducing household size by marrying off a daughter must be weighed against the costs associated with providing a dowry. Another economic motivation for marrying off a daughter may arise in bride price systems, where marriage involves a transfer from the groom's family to the bride's family, a practice common in many Sub-Saharan African contexts. Under economic hardship, bride price payments can function as a consumption smoothing strategy, allowing households to alleviate immediate financial constraints. Qualitative evidence from Zambia suggests that some parents and guardians view child marriage as an opportunity to benefit financially from bride price payments to ease household resource constraints and to support younger children (19). Likewise, empirical evidence from Tanzania shows that marriage payments constitute a major driver of child marriage (20), while more recent work provides broader empirical support for this relationship across across countries in Africa, South Asia and the Middle East (21; 22; 15) ." (see pp. 1-2)

4. **"may have interacted" [use the past tense because the study may be published several years after the pandemic]**

Thank you, we fully agree. We have now adjusted the whole paragraph as shown in the response below.

5. **Sentence unclear "You mean because of getting pregnant?" Also in the following sentence some mistakes. & Also because of the pandemic? Needs to clarify...**

Thank you for this comment and your careful review. We agree that there was a lack of clarity in our previous write-up. As Reviewer 2 raised a similar concern, we have rewritten the paragraph to improve clarity and coherence.

New version:
"Previous studies have extensively analysed how external economic shocks, particularly those arising from natural disasters, affect child marriage rates (7; 18; 24). However, far less is known about how country-specific marriage customs and gender norms moderate these effects. Some direct evidence on marriage responses to economic distress is provided by Chort et al. (25), who examine the interaction between income shocks and bride price norms in Turkey. Exploiting rainfall shocks as an exogenous source of income variation, they show that girls exposed to negative income shocks during adolescence face a significantly higher probability of being married before age 15 in provinces where bride

price is prevalent. These findings indicate that child marriage may function as a household coping strategy in response to economic shocks, even outside the poorest country contexts. However, as Haq et al. (26) [p. 9] caution, , "mixed studies combining qualitative and quantitative methods are needed to examine the effects of the pandemic on marriage," underscoring the need for integrative approaches that jointly consider the economic shock induced by the COVID-19 pandemic, marriage institutions, and gender norms." (see p.2)

6. **This is also unclear. Perhaps needs explaining more for the reader**

Thank you for this suggestion, we have decided to reformulate the sentence. You can see our improvements in the following revised paragraph:

"Building on this, our paper examines the links between pandemic-induced household income shocks and the probability of child marriage. We further investigate whether this association is moderated by cultural practices around marriage transfers and differs between bride price and dowry contexts. Specifically, we hypothesise that the pandemic would reduce the likelihood of child marriage in dowry-based societies, because brides' parents may face constraints in mobilising the resources required for dowry payments during periods of financial stress. Conversely, we hypothesise that the pandemic will increase the likelihood of child marriage in bride-price-based systems, as the brides' parents may use transfers from the groom's family as a means of consumption smoothing." (see p. 3)

7. **"they not only do..." or "they not only have..."? Unclear. Please check.**

Thank you for catching this. We have now corrected this as follows:

"Our study presents the first comparative analysis of India and Zambia. It combines quantitative regression methods with qualitative interviews to provide a comprehensive assessment of how the pandemic affected child marriage decisions. These two countries were selected because they represent distinct marriage systems. India is a dowry-based society, while Zambia is characterised by the practice of bride price. At the same time, both countries are located in regions with some of the highest child marriage rates worldwide." (see p. 3)

8. **Add reference to table 1 and adding whole numbers to have a full picture to both figures about child marriage trends.**

Thank you for this suggestion. We now explicitly reference Table 1 and 2 in the text when discussing child marriage rates in India and Zambia. In addition, we have added a short explanation of the overall trend, noting that although rates have declined, they remain high:

"Nevertheless as shown in Table 1, the overall child marriage decreased over the last 25 years, but remains high, meaning that 23.3% of all children were married under age in 2021. [...]
For Zambia, the currently available data indicate a downward trend in child marriage rates over the past decades. As shown in Table 2, the rate has decreased from 46.9% in 1993 to 29% in 2018." (see p. 12)

In addition, we have added table notes to both tables, now stating from which data source the numbers reported have been drawn from (namely, the Child Marriage Data Portal).

9. **Also realize that cultural norms are more "conservative" in rural areas than in urban ones.**

Thank you for pointing out that we missed an explanation about the cultural channels and on how norms can affect the correlates of child marriage. We decided to rephrase the paragraph and cited additional studies to include further evidence about this mechanism:

"Apart from this, the effect of poverty is particularly strong in rural areas (32). Al-Ridhwany et al. (29) confirm an urban-rural divide in child marriage rates in their study from northern Iraq. Corroborating this, several studies suggest that the urban–rural divide in child marriage also reflects differences in prevailing gender norms. Using Afrobarometer data from 34 African countries, Charles (37) shows that support for women's equal rights is systematically higher among urban residents and among individuals more exposed to extra-local cultural influences. Similarly, evidence from Demographic and Health Surveys across multiple sub-Saharan African countries indicates that, in most cases, rural residence is associated with a higher likelihood of justifying intimate partner violence against women, a commonly used proxy for conservative gender norms, although important cross-country variation exists (38)." (see pp. 5-6)

10. **Something is missing in this sentence, please check.**

Thank you for identifying this issue. We have corrected the sentence and have conducted an additional proofreading pass:

" *Yet, existing research rarely investigates economic stress by additionally considering the respective local culture context.*" (see p. 9)

11. **Also, geographically there is a spread between Africa and Asia**

We thank you for this helpful comment. In response, we have clarified the motivation for our country selection by emphasizing the comparative logic of studying similar pandemic-related economic shocks under different marriage institutions.

New version:
"The study focuses on two countries: India and Zambia. Menon (2020) estimates that 1.5 million girls in India are married before reaching adulthood, and that almost one in three girls are married before their 18th birthday in Zambia. These high prevalence rates make both countries relevant contexts for studying child marriage." (see p. 11)

12. This will have to add to the limitation of the study (linking to our small number of focus group interviews in Zambia)

Thank you for this important comment. We have added a limitation noting that the small number of focus group discussions in Zambia limits the depth of contextual insights:

"In addition, the relatively small number of focus group discussions conducted in Zambia limits the depth of contextual insights that can be drawn for this setting." (see p. 28)

13. and 14.

Thank you for pointing these out. We have corrected to "levels" and "linearly".

14. compared to how many assets in India?

Thank you for catching this. We agree that the interpretation of our previous descriptive statistics was not sufficiently clear and have therefore added: *"...their households owned, on average 6.7* ***out of a list of 14*** *assets." (see p.17)*

15. Not clear. From Zambia? And the difference with India?

Thank you for catching this. We have now carefully checked this whole section. We first describe descriptive statistics for the Indian sample, followed by a section focused on the Zambian sample. The sections are divided through subheadings "India" and "Zambia". However, we agree with you that it was sometimes confusing to know which sample we refer to, therefore, we have now added the respective country names to increase the readability for the descriptive statistics. We hope that this has helped making it easier to understand where the cited numbers come from.

16. This paragraph will improve if you try comparing with two numbers (for the two countries), rather than just citing one (for the benefit of the reader, even if one can go to the table). With these descriptions, it is NOT CLEAR which numbers are for which countries. Please review and add.

Thank you. We make comparisons between the descriptive statistics in both samples where we find interesting differences. As described in our response above, we hope that we have now improved readability of the paragraph by adding specific reference to the

country and sample the highlighted numbers refer to. We have also revised the text so that a more direct comparison becomes easier without looking at the table:

*"Overall household wealth in Zambia was also lower than in India, with an average of 4.7 (**compared to 6.7 in India) out of 14 assets** owned and living standard indicators achieved by the family." (see p. 18)*

17. **Who mentioned this comment?**

"Sometimes, what causes that [early marriage] is that there is no one to pay for her [adolescent girls'] school fee and she is just living at home. Then she will think of getting married instead of staying at home."

Thank you for the comment. We have now clarified that the statement was made by the mother of an adolescent girl in Mazabuka:

"... as expressed in the following statement by the mother of an adolescent girl in Mazabuka:" (see p. 23)

18. **This is already said above; see if it's possible to join both (or separating the second part of above and putting it here in the attending ceremony part.**

Thank you for the careful revision and suggestions for improved writing. We have revised the transition between the discussion of dowry reductions and wedding ceremony costs to clarify that both refer to the same underlying cost-reduction mechanism, thereby avoiding repetition.

New version:
"Together, these findings indicate that reductions in dowry demands and wedding ceremony costs reflect a single cost-reduction mechanism through which the pandemic altered marriage decisions. In addition to reduced dowry demands, the costs associated with wedding ceremonies emerged as a closely related factor. In India, wedding ceremonies often involve several hundred guests, and it is customary for the bride's family to bear this expense. However, our qualitative data highlighted that during the COVID-19 pandemic and the related contact restrictions, wedding ceremonies were significantly smaller, with only a few guests allowed to attend, and thus substantially cheaper." (see p. 24)

19. **and 20. To make it clearer, add "India" as a sub-title, so the reader will realize all that follows is for such country, and then below also add a subtitle for Zambia**

Thank you for this helpful suggestion. We have added separate subheadings for India and Zambia to clearly distinguish the country-specific analyses.

20. **Important to hypothesize that a pandemic may influence differently than with other disasters, given not only the economic crisis but also other aspects such as blockage, societal overall damage, etc.**

Thank you and we fully agree that the pandemic was a unique sort of an external shock, with some mechanisms likely differing from those of natural disasters. We have therefore added the following additional aspect to our discussion of limitations:

"*While our findings clearly differ from those reported in Corno et al. (105), it is important to note that the COVID-19 pandemic represents an unprecedented external shock that is not fully comparable to natural disasters. In particular, the prolonged economic consequences of lockdown policies likely imposed a distinct form of financial strain on households, differing from the strain typically observed during environmental or humanitarian disasters, which are often accompanied by substantial inflows of humanitarian aid and relief programmes. Moreover, our results indicated that social distancing policies were deliberately leveraged to reduce expenditures on daughters' weddings, a mechanism specific to the pandemic context and unlikely to generalise to other types of shocks."* (see pp. 27-28)

Reviewer 2

We are very pleased that you considered our study to be in line with the journal scope of interest. Thank you for your thorough review of our paper and for the suggestions for improvement as well as for identifying small typos and inconsistencies.

1. Missing citation for the first sentence and improved reference

Thank you for this comment. We agree that the original statement was not sufficiently specific. We have added a citation from the Girls Not Brides website and revised the beginning of the second sentence to improve clarity and readability.

New version:
"Child marriage is a global problem rooted in gender inequalities and the belief that the social status of girls and women is inferior to their male counterparts (Girls not Brides, 2025). Child Marriage is defined as "any formal marriage or informal union between a child under 18 and an adult or another child" (UNICEF, 2024)." *(see p. 1)*

2. Adding evidence/citations to the consequences of the pandemic on child marriage

Thank you for this valuable suggestion. Following your advice, we now explicitly cite Psaki et al. (2021), which was already included in our reference list but had not been cited in this paragraph. As highlighted by Psaki et al. (2021), economic factors and social norms are major drivers of child marriage. The pandemic, as an external shock to household

welfare, has therefore contributed substantially to increased economic hardship and heightened risk factors for child marriage.

3. **Vague and more citation needed in this section when discussing the economic shock on households and child marriage.**

Thank you for pointing this out and we fully agree that we need to better embed our arguments in existing empirical literature. We have therefore added the following additional paragraph and cite the additional papers listed below:

"Likewise, empirical evidence from Tanzania shows that marriage payments constitute a major driver of child marriage (20), while more recent work provides broader empirical support for this relationship across across countries in Africa, South Asia and the Middle East (21; 22; 15)." (see p. 2)

- *Psaki SR, Melnikas AJ, Haque E, Saul G, Misunas C, Patel SK, et al. What Are the Drivers of Child Marriage? A Conceptual Framework to Guide Policies and Programs. The Journal of Adolescent Health. 2021;69(6S):S13-22. Available from: https://www.sciencedirect.com/science/article/pii/S1054139X21004456.*
- *Corno L, Voena A, et al. Selling daughters: age of marriage, income shocks and the bride price tradition. Institute for Fiscal Studies; 2016.*
- *Kohno A, Techasrivichien T, Suguimoto 848 SP, Dahlui M, Nik Farid ND, Nakayama T. Investigation of the key factors that influence the girls to enter into child marriage: A meta-synthesis of qualitative evidence. Plos One. 2020;15(7):e0235959.*
- *Pope DH, McMullen H, Baschieri A, Philipose A, Udeh C, Diallo J, et al. What is the current evidence for the relationship between the climate and environmental crises and child marriage? A scoping review. Global Public Health. 2023;18(1):2095655.*

4. Unclear sentence (Page 2) & vague - what sort of marriage trends and figures?

We agree with the reviewer's assessment. In response, we have rewritten the entire paragraph, also addressing several related points raised by Reviewer 1. We hope that this revised discussion more clearly articulates the nexus between child marriage and gender norms and strengthens the academic contribution of the manuscript.

New version:
"Previous studies have extensively analysed how external economic shocks, particularly those arising from natural disasters, affect child marriage rates (7; 18; 24). However, far less is known about how country-specific marriage customs and gender norms moderate these effects. Some direct evidence on marriage responses to economic distress is provided by Chort et al. (25), who examine the interaction between income shocks and bride price norms in Turkey. Exploiting rainfall shocks as an exogenous source of income

variation, they show that girls exposed to negative income shocks during adolescence face a significantly higher probability of being married before age 15 in provinces where bride price is prevalent. These findings indicate that child marriage may function as a household coping strategy in response to economic shocks, even outside the poorest country contexts. However, as Haq et al. (26) [p. 9] caution, , "mixed studies combining qualitative and quantitative methods are needed to examine the effects of the pandemic on marriage," underscoring the need for integrative approaches that jointly consider the economic shock induced by the COVID-19 pandemic, marriage institutions, and gender norms." (see p.2)

5. Awkward sentence – where is this based? (Page 4)

Thank for catching this. We have decided to further elaborate on the evidence given by Envaladu et al. (2025) and have edited the paragraph as follows:

"Apart from this, Envuladu et al. (32) document a significant positive association between low parental education and increased risk of underage marriage among girls, suggesting that parental educational attainment plays a vital role in shaping marriage-related decisions (35)." (see p. 5)

6. Avoid the word "poorer" (Page 4, high frequency of child marriage in some regions)

Thank you for raising this important point. We fully agree and have replaced the term with "regions affected by instability and low income". We chose this formulation because political and economic instability is a key driver of child marriage and more accurately reflects the underlying mechanisms discussed in the paper.

7. Whose awareness? (Page 5, study by Fattah and Camellia 2022) linked to the following comment about missing evidence (Page 6)

Thank you for this suggestion. As the paragraph was somewhat unclear, we have revised it to explicitly state that the discussion of social norms and the status of young girls is grounded in the work of Fattah and Camelia (2022).

New version:
" Fattah and Camelia used a mixed-methods design in rural Bangladesh and reported high levels of awareness among participants regarding the negative consequences of child marriage (45). Their study sample was composed of socio-demographically diverse, male and female participants with varying levels of education and self-reported household economic status, ranging from extreme poverty to wealth. The authors further highlighted that participants perceived early marriage as a means of protecting family reputation, as

it was believed to prevent social stigma in cases where a young girl experienced sexual assault or harassment." (see p. 6)

7. Citation fix (Page 6)

Thank you for noting this mistake. The citation has been corrected and changed to the Vancouver style.

8. Avoid pronouns (Page 7)

Thank you for highlighting this. We agree that pronouns should be avoided in scientific writing and have revised the text accordingly.

9. Avoid the term "developing countries" (Page 7)

Thank you for this suggestion. We replaced the term with "low- and middle-income countries".

10. Evidence? (Page 7)

Thank you and we have toned down this statement as follows:

"Only few empirical studies have evaluated the impacts of the COVID-19 pandemic on child marriage rates." (see p. 8)

11. Adding A and not the (Page 8)

Thank you for pointing this out. We have made the suggested change.

12 + 13. What is the focus of the paper of Brandl and Colleran (2024) and how do we extend on it (Page 8)

Thank you for raising this concern. We have now clarified that Brandl and Colleran (2024) provide an important contribution by synthesising different strands of the child marriage literature and highlighting links between economic hardship and child marriage. However, as their study is a literature review, it does not provide original empirical evidence, which distinguishes our contribution. However, to avoid implying that the Brandl and Colleran paper has specific shortcomings, we have also changed the following sentence to improve the entire paragraph.

"Brandl and Colleran provide a theoretical link between marriage payments and marriage patterns; however, their analysis remains largely conceptual, as it does not provide quantitative evidence to support the proposed framework (70). Our paper builds on their

contributions by offering comparative evidence from India and Zambia. These two contexts are characterised by contrasting marriage institutions, namely dowry and bride price, which allows us to examine how these mechanisms interact. We provide both statistical estimates and in-depth contextual understanding to uncover the micro mechanisms that drive child marriage under pandemic related stress, offering a more comprehensive perspective than previous research. The subsequent section introduces our theoretical framework and outlines the hypotheses guiding our analysis." (see p. 9)

14. Gendered version of headmaster (Page 11)

Thank for this comment. After carefully checking our documents, the headmaster were female and male, we clarified it in our manuscript:

"School-attending girls were recruited directly within schools where female and male headmasters and class teachers provided a list of all girls aged 13-18 years." (see p. 13)

15. Explain the procedure of random walk when doing interviews and unclear sentence. (Page 11)

Thank you for this comment and we agree that the previous version of the manuscript was lacking clarity here. We expanded the methodological description to clarify the procedure. We now explain that data collectors began at a randomly selected starting point within each community and approached every *n*th household following a predefined route until the target sample size was reached.

New version:
"This recruitment process was further facilitated by local community leaders and Anganwadi workers using a random walk procedure, where data collectors began at a randomly selected starting point within each community and then approached every third household, defined as selecting one household after systematically skipping the next two households, following a predefined route until the target sample size was reached."
(see p.13)

16. Explain how you selected data in Zambia, unclear sentence (Page 13)

Thank you for this comment. We have revised the sentence to clearly describe the procedure. Specifically, we explain that 100 schools across the districts of Kalomo, Mazabuka, and Monze were approached, and that school administrators provided information on catchment areas to identify communities where age-eligible girls were likely to reside.

New version:
"To identify eligible girls, we approached 100 schools across the three districts of Kalomo, Mazabuka, and Monze. School administrators provided information on the schools' catchment areas and the surrounding communities from which enrolled students typically come. From these, we randomly selected four catchment areas to which enumerator teams were then deployed to recruit age-eligible girls through a random walk procedure, following a similar logic as in India (see above). Our final sample consisted of 1615 girls who were recruited within 92 school clusters." (see p. 13)

17. Change couls to could

Thank you for noting this typo. We have corrected the error and have carefully reviewed the manuscript for additional spelling mistakes.

18. More generally, I would like to see the conceptual framework referred to at the onset of the paper referred to in greater depth in the discussion.

Thank you very much for highlighting this very important point. We agree that our conceptual framework should have been integrated more prominently throughout our manuscript. In line with your suggestion, we have made the following changes:

- Introduction section: We now already previous the conceptual framework early on in the introduction section, where we first describe our two hypotheses:
  "Specifically, developing a conceptual framework of child marriage, which integrates factors on the individual, socio-demographic, and institutional level, *we hypothesise that the pandemic would reduce the likelihood of child marriage in dowry-based societies, because brides' parents may face constraints in mobilising the resources required for dowry payments during periods of financial stress. Conversely, we hypothesise that the pandemic will increase the likelihood of child marriage in bride-price-based systems, as the brides' parents may use transfers from the groom's family as a means of consumption smoothing." (see p. 3)*

- Theoretical framework section: In this section, we now introduce the framework at the beginning of the chapter, rather than at the end and have made the following additions:
  "To guide our analysis, we create a conceptual framework as shown in Figure 1. [....] Below, we introduce our conceptual framework by drawing on evidence on the correlates of child marriage at the individual, socioeconomic, and institutional level as well as links between economic shocks and child marriage. We then delineate our two guiding hypotheses on the differential effects of economic shocks in bride price and dowry systems." (see p. 4)

- We have also moved Figure 1 up, so that it is introduced not at the end of the chapter but early on.

- Discussion section: Following your helpful guidance, we now also reference our conceptual framework in the discussion section by clearly stating which predictions of the conceptual framework our empirical analysis did not support:

  "Finally, with regards to the impact of economic shocks, our analysis did not support the hypothesis ***derived from our conceptual framework*** *that child marriage rates would likely decrease in consequence of the economic shock caused by the COVID-19 pandemic. " (see p. 26)*

  "But while the COVID-19-related economic shock played a crucial role in the family's financial situation, it did not lead to an increase in child marriages ***as predicted by our conceptual framework****." (see p. 27)*

  "While our findings clearly differ from those reported in Corno et al. and from the predictions made by our conceptual framework, it is important to note that the COVID-19 pandemic represents an unprecedented external shock that is not fully comparable to natural disasters. In particular, the prolonged economic consequences of lockdown policies likely imposed a distinct form of financial strain on households, differing from the strain typically observed during environmental or humanitarian disasters, which are often accompanied by substantial inflows of humanitarian aid and relief programmes. Moreover, our results indicated that social distancing policies were deliberately leveraged to reduce expenditures on daughters' weddings, a mechanism specific to the pandemic context and unlikely to generalise to other types of shocks. Against this background, our conceptual framework cannot be validated in the specific context of the COVID-19 pandemic." (see pp. 27-28)
